# Heterologous Expression and Biochemical Characterization of a New Chloroperoxidase Isolated from the Deep-Sea Hydrothermal Vent Black Yeast *Hortaea werneckii* UBOCC-A-208029

**DOI:** 10.1007/s10126-023-10222-7

**Published:** 2023-06-24

**Authors:** Bastien Cochereau, Yoran Le Strat, Qiaolin Ji, Audrey Pawtowski, Ludovic Delage, Amélie Weill, Lisa Mazéas, Cécile Hervé, Gaëtan Burgaud, Nina Gunde-Cimerman, Yves François Pouchus, Nathalie Demont-Caulet, Catherine Roullier, Laurence Meslet-Cladiere

**Affiliations:** 1 Univ Brest, INRAE, Laboratoire Universitaire de Biodiversité et Ecologie Microbienne, F-29280 Plouzané, France; 2 Institut des Substances et Organismes de la Mer, Nantes Université, ISOMER, UR 2160, F-44000 Nantes, France; 3 Integrative Biology of Marine Models (LBI2M), UMR8227, Station Biologique de Roscoff (SBR), CNRS, Université, 29680 Roscoff, Sorbonne France; 4 Molecular Genetics and Biology of Microorganisms, Dept. Biology, Biotechnical Faculty, University of Ljubljana, Ljubljana, Slovenia; 5 INRAE, University of Paris, UMR ECOSYS, INRAE, Université Paris-Saclay, 78026 Versailles, AgroParisTech France; 6 Univ Brest, UBO Culture Collection (UBOCC), F-29280 Plouzané, France

**Keywords:** Marine fungi, Black yeast, Halogens, Enzymes, Vanadium chloroperoxidase

## Abstract

The initiation of this study relies on a targeted genome-mining approach to highlight the presence of a putative vanadium-dependent haloperoxidase-encoding gene in the deep-sea hydrothermal vent fungus *Hortaea werneckii* UBOCC-A-208029. To date, only three fungal vanadium-dependent haloperoxidases have been described, one from the terrestrial species *Curvularia inaequalis,* one from the fungal plant pathogen *Botrytis cinerea*, and one from a marine derived isolate identified as *Alternaria didymospora*. In this study, we describe a new vanadium chloroperoxidase from the black yeast *H. werneckii*, successfully cloned and overexpressed in a bacterial host, which possesses higher affinity for bromide (K_m_ = 26 µM) than chloride (K_m_ = 237 mM). The enzyme was biochemically characterized, and we have evaluated its potential for biocatalysis by determining its stability and tolerance in organic solvents. We also describe its potential three-dimensional structure by building a model using the AlphaFold 2 artificial intelligence tool. This model shows some conservation of the 3D structure of the active site compared to the vanadium chloroperoxidase from *C. inaequalis* but it also highlights some differences in the active site entrance and the volume of the active site pocket, underlining its originality.

**Supplementary Information:**

The online version contains supplementary material available at 10.1007/s10126-023-10222-7.

## Introduction

**Fig. 1:**
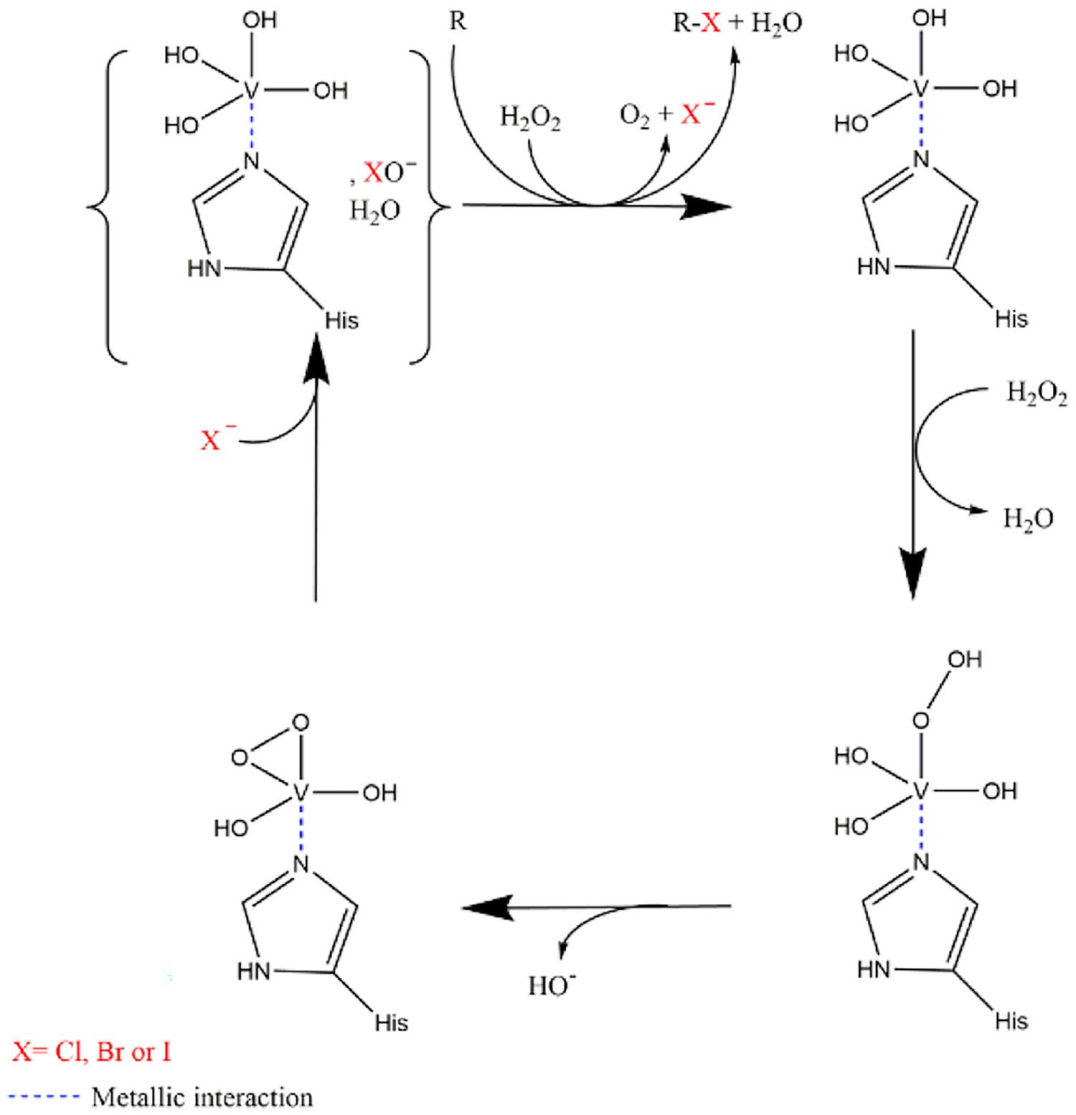
Vanadium-dependent haloperoxidase enzymatic mechanism (Cochereau et al. 2022)

Halogenated natural products (NPs) in nature are characterized by a wide variety of bioactivities. As specialized metabolites, they can be involved in different mechanisms from communication to chemical defense. Halogenated NPs represent approximately 10,000 compounds which harbor fluorine, chlorine, bromine, or iodine on their structures (Cochereau et al. 2022). Among these, 1003 compounds are produced by fungi, and our previous work has shown the potential of marine species in this area (Roullier et al. 2016). Halogenated NPs have attracted much attention, especially in the marine environment where chloride and bromide concentrations are 20 to 60 times higher than in the terrestrial environment (Dickson and Goyet 1994; Gribble 2010). In nature, to produce these halogenated compounds, organisms include genes in their genome encoding different halogenating enzymes (Butler and Sandy 2009). Nowadays, a catalog of halogenating enzymes has been drawn up from different clades of organisms, i.e., flavin-dependent halogenases (FDH), haloperoxidases, non-heme iron-dependent halogenases, or SAM-dependent halogenases (Agarwal et al. 2017). Specifically, the haloperoxidase family includes heme-dependent haloperoxidases (hHPOs) and vanadium-dependent haloperoxidases (vHPOs). Vanadium-dependent haloperoxidases are metalloenzymes which stabilize vanadate (VO_4_^−^) in their active site. Vanadate can then react with hydrogen peroxide (H_2_O_2_) to oxidize halides (Cl^−^, Br^−^ and I^−^ but not F^−^ due to its high electronegativity) and form the corresponding hypohalous acid (OH^−^X^+^). This reactive species is then released in the proximal environment where it can react with a non-specific substrate to form halogenated metabolites (Leblanc et al. 2015; Cochereau et al. 2022) (Fig. 1). vHPOs are named according to the most electrophilic halogen they can oxidize: (i) a chloroperoxidase can oxidize chloride, bromide or iodide; (ii) a bromoperoxidase can oxidize bromide or iodide, and (iii) an iodoperoxidase can only oxidize iodide (Agarwal et al. 2017). vHPOs were first described in the alga *Ascophyllum nodosum* in 1984 (Vilter 1984). To date, thirteen vHPOs have been described from algae: seven are from the brown algae *Laminaria digitata*, *Pelvetia canaliculata*, *Laminaria hyperborean*, *Laminaria saccharina*, and *A. nodosum* (Almeida et al. 2000, 2001; Colin et al. 2005; Wischang et al. 2012). The remainder are from the red algae *Corallina officinalis*, *Corallina pilulifera*, *Gracilaria changii*, *Ceramium rubrum*, and *Laurencia nipponica* (Krenn et al. 1987; Carter et al. 2002; Ohshiro et al. 2004; Baharum et al. 2013; Kaneko et al. 2014). Interestingly, all the vHPO described in algae are bromoperoxidases (vBPOs) or iodoperoxidases (vIPOs). Subsequently, additional vHPOs were identified in members of the bacterial domain (24 proteins) (Agarwal et al. 2017), for example in *Streptomyces* sp. (Mckinnie et al. 2018) or in marine bacteria such as *Zobellia galactanivorans* (Fournier et al. 2014). In fungi, only three vHPOs have been reported so far. One was structurally described from the terrestrial fungus *Curvularia inaequalis* (Messerschmidt and Wever 1996), another has been described in terms of its primary structure and has been characterized biochemically. It is the chloroperoxidase from the marine fungus *Embellisia didymospora*, isolated from seawater samples of the Adriatic Sea, recently renamed *Alternaria didymospora* (Barnett et al. 1998). The third one has bromoperoxidase activity and is detected in the lichen *Xanthoria parietina* with a protein of 65 kDa but, it is not known whether this belongs to the fungal or the algal part of this organism (Plat et al. 1987). Vanadium haloperoxidases present several advantages: (i) they allow the halogenation of many different electron-rich compounds because the halogenation is unspecific, (ii) these enzymes are very stable in organic solvents, and (iii) some vHPOs can be regioselective (Agarwal et al. 2017; Younes et al. 2019; Chen et al. 2022). Consequently, they represent very attractive alternatives to usual synthetic reagents for halogenation from the perspective of greener chemistry (Höfler et al. 2019). For example, the chloroperoxidase from *C. inaequalis* is widely studied today for its biocatalytic capacities (Höfler et al. 2019; Younes et al. 2019). Because vHPOs are interesting in the biocatalysis field, and because fungi have been very poorly investigated in this area, we recently showed their potential by analysis of 82 genomes showing 105 sequences matching the vHPO genes in the Mycocosm database (Cochereau et al. 2022). Regarding the interest for this type of enzymes, one strain from our collection of marine fungi was further investigated from this perspective.

In this study, we report the discovery and the biochemical characterization of the second vHPO from a marine fungus. We describe a vanadium-dependent chloroperoxidase from *Hortaea werneckii* (Hw, UBOCC-A-208029), an impressive extremely halotolerant black yeast collected from the Rainbow hydrothermal vent field (Gunde-Cimerman et al. 2009). *H. werneckii* (*Capnodiales*, *Ascomycota*) is a black yeast, globally present in hypersaline environments (Zalar et al. 2019; Romeo et al. 2020). *H. werneckii* grows like yeast cells in liquid culture media, but it can switch to filamentous growth on solid substrates and into meristematic clumps at extreme conditions (Kogej et al. 2007). This polymorphic yeast-like melanized fungus was originally known as the causative agent of human tinea nigra, a characteristic type of colonization of salty human hands (de Hoog and Guého 2010). Here, the recombinant *Hw*vCPO was successfully overexpressed in *Escherichia coli* by co-expression with chaperone proteins to optimize protein folding. Finally, we describe the biochemical and enzymatic properties of this novel enzyme.

## Materials and Methods

### Fungal Strains

*Hortaea werneckii* UBOCC-A-208029 was collected from the Rainbow hydrothermal vent field, in the mid-Atlantic ridge in 2007 and is part of the UBO Culture Collection (UBOCC) of the University of Brittany (Burgaud et al. 2010). This isolate was cultivated on potato dextrose agar (PDA) media (BD Difco™ dehydrated culture media: potato dextrose agar; 39 g/L), minimal media (MM, glucose 5 g/L, KH_2_PO_4_ 1 g/L, KCl 0.5 g/L, NaNO_3_ 2 g/L, MgSO_4_, 7H_2_O 2.5 g/L, TE solution 2 mL/L); Czapek media (CZA, glucose 16 g/L, yeast extract 5 g/L, K_2_HPO_4_ 1 g/L, Czapek concentrate 10 mL/L) and Wickerham (W, glucose 10 g/L, peptone 5 g/L, yeast extract 3 g/L, malt extract 3 g/L). MM, CZA, and W solid media contain 20 g/L of agar. Culture media were supplemented with sea salts (3%) (Sigma-Aldrich) with or without the addition of halogenated salts (10%) (NaF 0.02 g/L, KBr 0.09 g/L, and KCl 0.6 g/L). Trace elements (TE) solution contained (g/L): citric acid 5 g, ZnSO_4_·7H_2_O 5 g, FeSO_4_·7H_2_O 4.75 g, Fe(NH_4_)_2_(SO_4_)_2_·6H_2_O 1 g, CuSO_4_·5H_2_O 0.25 g, MnSO_4_·H_2_O 0.05 g, H_3_BO_4_ 0.05 g, and NaMoO_4_·2H_2_O 0.05 g. Czapek concentrate solution contains (g/L): NaNO_3_ 30 g, KCl 5 g, MgSO_4_·7H_2_O 5 g, FeSO_4_·7H_2_O 0.1 g, ZnSO_4_·7H_2_O 0.1 g, and CuSO_4_·5H_2_O 0.05 g.

### Bacterial Strains

Recombinant plasmid was maintained in *E. coli* JM109 (Promega) before being used to transform *E. coli* BL21(DE3) for the overexpression of recombinant proteins (Invitrogen, ThermoFisher Scientific). *E. coli* BL21(DE3) was first-transformed with plasmid pKJE7 from Takara Bio which permits co-expression of protein chaperones DnaK, DnaJ and GRP E (Takara Bio, Kusatsu, Japan). These cells were used for the overexpression of recombinant proteins. Bacteria were cultivated at 37 °C and 18 °C on Lysogeny Broth solid medium (LB) (0.1% peptone, 0.05% yeast extract, 0.1% sodium chloride, 0.1% agar) or Lysogeny Broth low salt liquid medium (LB Low Salt) (0.1% peptone, 0.05% yeast extract, 0.05% sodium chloride) or Terrific Broth liquid medium (TB) (1.2% peptone, 2.4% yeast extract, 0.4% glycerin 87%, 10% phosphate buffer pH 7.5 10x). Culture media were supplemented with chloramphenicol (20 µg/mL) and ampicillin (50 µg/mL) for selection.

### Phylogenetic Analysis

The selected set of vHPO proteins was retrieved from several NCBI Blastp searches. *Hw*vCPO sequence was used to identify homologous sequences against the fungi and the bacterial RefSeq databases. In addition, eukaryotic sequences, absent from Refseq, were found by querying the nr database. Finally, the crystallized vHPO sequences and their closest counterparts plus three acid phosphatases constituting an outgroup were added to the dataset (see Supplementary Table 1 for complete information). A total of 105 sequences were loaded into a “A la carte” pipeline analysis on the “NGPhylogeny.fr” website. The protein sequences were aligned under default parameters using MAFFT tool resulting in 1200 amino acid positions. A selection of 469 informative positions was obtained by trimAl. The maximum likelihood phylogenetic tree construction was carried out using default parameters of the PhyML-SMS tool allowing the best substitution model selection (WAG + G + I + F). The resulting Newick file was formatted and annotated using the MEGA v10.1.1 software to show the major supported nodes and groups (100 bootstrap replicates; support value > 80%) leading to the corresponding simplified phylogram. A limited number of fungal sequences were deliberately taken (limited to about half of the total) to avoid biasing the choice of informative positions (IPs) in favor of these same fungi by the IP selection tool.

### mRNA Extraction

Extractions were performed using the NucleoSpin RNA Plus from Macherey Nagel according to the manufacturer’s protocol with 30 mg of yeast. mRNAs were stocked at − 80 °C until analysis. mRNA concentrations were estimated with a NanoDrop 2000.

### RT-qPCR Analysis

Production and expression of the vHPO mRNA from *H. werneckii* UBOCC-A-208029 were analyzed in four different culture media as described previously (MM, PDA, W, and CZA) with added sea salts and with or without halogens supplementation (Cf. 2.1). Analyses were performed using a CFX96 BioRad^®^. This device allowed the automated calculation of mRNA concentrations using a pre-established standard range (0.5 to 100 ng/µL). mRNA extractions were carried out in triplicate. Two technical replicates were also prepared for each biological replicate. Results were normalized with mRNA of a reference gene, the phosphofructokinase, involved in glycolysis (GenBank ID: OTA38163.1) (Gillot et al. 2017).

### Cloning of *H. werneckii* Vanadium-dependent Chloroperoxidase in *E. coli*

A putative vHPO-encoding gene was first identified in the related isolate Hw EXF-2000, obtained from marine solar salterns, for which whole genome sequence is available in MycoCosmo platform (www.ex-genebank.com) (Lenassi et al. 2013). Hw EXF-2000 was used as a model to design specific primers to amplify homologous sequences from the isolate UBOCC-A-208029 obtained from deep-sea hydrothermal vent samples (Burgaud et al. 2010). The vHPO gene from *H. werneckii* UBOCC-A-208029 was amplified using mRNA extracted using the NucleoSpin^®^ RNA Extraction kit (Macherey–Nagel, Hoerdt, France). cDNA was obtained using the Invitrogen™ ThermoScript™ RT-PCR System for First-Strand cDNA Synthesis kit following the manufacturer’s recommendations (Invitrogen, Carlsbad, CA, USA). Amplification of the specific cDNA encoding the vHPO was achieved using specific primers: *Hw_vHPO_Fw* (5′-ATGATTCCACTTCACCAGCG-3′) and *Hw_vHPO_Rev* (5′-TTAGGGCTTTCCAAGGCGTCCACACCTATGCCTG-3′). Once cDNA was synthesized, PCR amplification was carried out with the Phusion^®^ HF DNA Polymerase kit (BioLabs) using 30 μL distilled water, 10 μL Phusion Buffer, 1 μL DNTP 10 mM, 0.5 µM Primer vHPO Forward (Eurofins Genomics), 0.5 µM Primer vHPO Reverse (Eurofins Genomics), 1.5 μL DMSO 100%, and 0.5 μL Phusion Polymerase for 2–3 ng cDNA. The amplification consisted of an initial denaturation step at 94 °C for 2 min followed by 5 iterations of 1 min 98 °C, 1 min 60 °C, and 2 min 30 s 72 °C. Then 30 iterations of 1 min 98 °C, 1 min 65 °C and 2 min 30 s 72 °C with a final extension step of 10 min 72 °C. The amplified fragments were extracted and purified from a 1% agarose gel using the NucleoSpin Gel PCR Clean-up kit (Macherey–Nagel, Hoerdt, France). For protein overexpression, the vHPO gene was ligated into previously linearized (*PstI* and *HindIII,* Promega) pQE-81L vector (Qiagen, Hilden, Deutschland) with In-Fusion^®^ HD Cloning kit (Takara Bio, Kusatsu, Japan) (primers for cloning: *Hw_IF-fw*: 5′-TACCCCGGGTCGACCTGCAGATGATTCCACTTCACCAGCGTCC3-′ and *Hw_IF_Rev*: 5′-TCAGCTAATTAAGCTTTAGGGCTTTCCAAGGCGTCC-3′). The ligated plasmid was used to transform BL21 (DE3) + pKJE7 strain, generating strain BL21 (DE3) + pKJE7 + vHPO_Hw.

### Expression and Purification of the Recombinant vHPO

For protein expression, the recombinant strain BL21(DE3) + pKJE7 + vHPO_Hw was grown in 100 mL LB low salt medium containing 50 µg/mL ampicillin and 20 µg/mL chloramphenicol at 180 rpm O/N at 37 °C. Subsequently, the preculture was used to inoculate 500 mL of TB containing 50 µg/mL ampicillin and 20 µg/mL chloramphenicol at 0.05 OD_600nm_ (UV-2401 PC, Shimadzu). The culture was grown under agitation at 37 °C. Chaperone expression was induced when the OD_600nm_ reached approximately 0.1, by the addition of 0.5 mg/mL of L-arabinose (final concentration). The expression of recombinant enzyme was induced by adding Isopropyl-β-D-thiogalactopyranoside (IPTG) to a final concentration of 0.25 mM between OD_600nm_ of 0.6–0.8. After induction, the culture was grown under agitation at 20 °C for 24 h. Cells were then collected by centrifugation at 8150 g for 15 min and resuspended in Tris/H_2_SO_4_ pH 8.1 0.1 M. Cells were broken by sonication (10 cycles of 10 s with 1 min rest on ice between each cycle, power 40%). Lysates were centrifuged for 15 min at 20,124 g at 4 °C and supernatant was collected and frozen at − 20 °C. Protein purification was performed using an Äkta Avant system at 20 °C (GE Healthcare, Chicago, IL, USA) with protein detection by UVs absorption at 280 nm. His-Tagged proteins were purified on immobilized nickel (Ni) tetradentate absorbent (NTA) medium, using a HisTrap FF 5 mL column (GE Healthcare). Chromatographic methods used (Buffer A: Tris/H_2_SO_4_ pH 8.1 0.1 M, 10 mM Imidazole and Buffer B: Tris/H_2_SO_4_ pH 8.1 0.1 M, 500 mM Imidazole). An isocratic gradient (0–100%) of buffer B was used to elute the protein from the HisTrap FF column and fractions were collected every 2 mL. The presence of the recombinant protein was immunorevealed in collected fractions using an antibody raised against His6-tag (Monoclonal Anti-polyHistidine − Peroxidase antibody produced in mouse A7058, Sigma–Aldrich, Saint-Louis, MO, USA). Fractions were dialyzed, pooled and concentrated using Amicon^®^ Ultra-15 Centrifugal Filter Devices 30 KDa (Merck Millipore, Burlington, Massachusetts, USA). 2 mL of concentrated proteins were then further purified using gel filtration on GF HiLoad Superdex 200 prep-grade column (GE Healthcare). Two column volumes of Tris/H_2_SO_4_ pH 8.1 0.1 M were used to equilibrate the column. One column volume was used to elute proteins. The fractions were then tested in enzymatic assays. The final concentration of purified protein was estimated to be around 5 mg/mL (by BCA assay) and aliquots of recombinant proteins were stored at − 20 °C.

### SDS-PAGE and Western-Blot

The presence and size of recombinant protein were checked on 12% SDS-PAGE and Western-Blot to validate fully expressed protein. The protein samples were loaded with Laemmli 4X (60 mM Tris–HCl pH 6.8, 10% glycerol, 2% SDS, 5% bromophenol Blue, 5% β- mercaptoethanol). A preliminary step of heating at 100 °C during 10 min was done before electrophoresis. The proteins were visualized by staining the gel with 0.2% Coomassie blue solution for 30 min and then destaining in a 20% ethanol and 10% acetic acid solution overnight, or transferred to Bio-Rad nitrocellulose membrane using a Trans-blot system (Bio-Rad) for Western blotting.

For Western-Blot, Nitrocellulose membranes were immersed in Tris-Buffer-Saline pH 7.6 0.1% Tween containing 5% milk for 30 min., then immersed in TBS-Tween-5% milk containing anti-his tag antibody (Monoclonal Anti-polyhistidine-Peroxidase antibody produced in mouse A7058, Sigma-Aldrich, Saint-Louis, MO, USA) at 1/5000 dilution for 45 min. Membrane was washed three times for 5 min in TBS-Tween. Detection was carried out using ECL™ Prime Western Blotting System (Cityva life sciences, Marlborough, USA). The protein marker used was Precision Plus Protein™ Prestained Standards all blue (BioRad).

### Enzymatic Assays

#### Thymol Blue Assay

Thymol blue (TB, pKa = 8.9, λ_max_ = 430 nm) assay was used to determine enzymatic activity regarding bromide and iodide (Verhaeghe et al. 2008). It is based on the formation of dibromothymolsulfonphthalein (TBBr_2_, pKa = 7.2; λ_max_ = 620 nm) or diiodothymolsulfonphthalein (TBI_2_, pKa = 7.3; λ_max_ = 620 nm) at pH 8 or 7 and can be observed by a color change of the reaction medium from yellow to blue/green. For BPO reaction, the medium contained 100 mM phosphate buffer pH 8.0, 100 µM thymol blue, 10 mM NaBr, 0.25 µg/mL enzyme, and 0.1 mM H_2_O_2_ complemented with ultrapure water. For IPO reaction, the medium contained 100 mM phosphate buffer pH 8.0, 100 µM thymol blue, 1.5 mM NaI, 0.25 µg/mL enzyme, and 0.35 mM H_2_O_2_ complemented with ultrapure water. Final volume was 50 µL. The stock solution of thymol blue (TB) 1 mM was prepared in H_2_O/DMSO (4:1), and the final DMSO content never exceeded 2%. Reactions were performed at 20 °C. The positive control was ZgIPO1 iodoperoxidase from *Z. galactanivorans* (Fournier et al. 2014) or the native *Ascophyllum nodosum* bromoperoxidase I (Hartung et al. 2008). The negative controls were buffer and protein crude extract of BL21 + pKJE7 culture.

##### o-Dianisidine PAGE Assay

The electrophoresis of pure proteins was carried-out on non-denaturing acrylamide gel (10%). Native loading buffer (200 mM Tris–HCl pH 6.8, 40% (v/v) glycerol, 0.2% (m/v) bromophenol blue in pure H_2_O Milli-Q^®^) was used for electrophoresis. Native migration buffer without SDS was used for migration (Tris buffer 25 mM pH 8.3, glycine 192 mM). The *o*-dianisidine PAGE assay measures the halogenation of *o*-dianisidine by vHPO with chloride, bromide or iodide. The production of halogenated *o-*dianisidine causes a modification of the absorption properties of the molecule with the appearance of a pink-brown coloration observable on the native gel (Jordan and Vilter 1990). Solution 1 contained *o*-dianisidine (100 mM in 1 mL of acetic acid) in 100 mM phosphate buffer pH 7.4 (final volume 100 mL). Solution 2 contained 5 mM H_2_O_2_. After the native electrophoresis, the gel was first incubated in 50 ml of solution containing 100 mM phosphate buffer pH 7.4, 4.5 mL solution 1, 1 mM KI, or 2 mM KBr or KCl in ultrapure water, for 1 h with stirring at 20 °C. Then, the gel was transferred to 50 mL of solution 2 for either 1 h. For iodoperoxidase (IPO) reaction, or overnight for BPO and CPO reactions, then subsequently washed with 50 mL of ultrapure water to stop the reaction. Peroxidase activity was checked by omitting halogen from the first incubation step. The positive control used *Zg*-vIPO1 from *Z. galactanivorans* or the native *Ascophyllum nodosum* bromoperoxidase I (Hartung et al. 2008; Fournier et al. 2014)*.* In the negative control, the vHPO protein/enzyme was replaced by water. 10 µL of purified *Hw*vCPO (5 mg/ml) were used for this assay.

#### Monochlorodimedone Assay

An MCD assay was used to determine enzymatic activity and specificity. This assay monitors the enzymatic conversion of monochlorodimedone (MCD) (ε = 19.9 mM^−1^.cm^−1^) by vHPO in dichlorodimedone or bromochlorodimedone (ε = 0.2 mM^−1^.cm^−1^). A decrease in the absorption at 290 nm reveals the enzymatic activity and is measured throughout the assay using a UV spectrophotometer (UV-2401 PC, Shimadzu). The Kinetics of the reaction were evaluated by measuring absorption every 0.5 s for 180 or 490 s. The following components were then added to the UV cuvette: 50 mM Buffer (pH 4 to 9), 50 µM MCD, various concentrations of KCl 2 M or KBr 2 mM (for constant calculations) or 200 mM KCl or 200 µM KBr (for pH assessment), 2 µL enzymatic solution (2.5 mg/mL), 10 µM Na_3_VO_4_ and various amounts of distilled water to obtain a final volume of 900 µL. Then 100 µL of H_2_O_2_ 12 mM were added to start the reaction. A negative control was used to confirm the enzymatic activity by replacing the enzyme solution with buffer. A positive control reaction was also prepared using the *Ascophyllum nodosum* vBPO (Hartung et al. 2008). Enzymatic activity calculations were carried out using the RStudio software by calculating non-linear regression (Marasović et al. 2017). To calculate enzymatic parameters, increasing amounts of KCl (20 to 500 mM), KBr (5 to 500 µM), and H_2_O_2_ (1 to 500 µM) were used. All reactions were performed in triplicate at 20 °C.

### Tolerance to Solvents Assay

MCD assays were also carried out to test the organic solvent tolerance of the *Hw*vCPO protein by replacing distilled water with a mixture of H_2_O/solvent (50:50 v/v). Solvents tested were acetonitrile (ACN), methanol (MeOH), N,N-dimethylformamide (DMF), dimethylsulfoxide (DMSO), and ethanol (EtOH). The final proportion of each solvent in the enzymatic reaction was 39.3%, following the addition of other solutions added in the reaction, i.e., 50 mM of buffer pH 7, 50 µM of MCD, 100 µM of KBr, 2 µL of *Hw*vCPO (2.5 mg/mL), 10 µM of Na_3_VO_4_, and 1.2 mM of H_2_O_2_; resulting in 786 µL of H_2_O/solvent (50:50 v/v) in the final reaction volume of 1 mL. Blanks were prepared without MCD for each solvent system, and the negative control by replacing the enzyme solution with distilled water. All reactions were performed in triplicate. Differences between groups were determined using one-way analysis of variance (one-way ANOVA) and post-hoc LSD tests, after verifying (i) the normality distribution with the Kolmogorov–Smirnov normality test and (ii) the equality of variances with a Bartlett test.

### Homology Model Building

AlphaFold 2 artificial intelligence program was used to build models for *Hw*vCPO. AlphaFold is based on predicted Local Distance Difference Test (pLDDT) to estimate model confidence on a scale from 0 to 100 (Mariani et al. 2013). pLDDT > 90 are expected to be modelled to high accuracy and can be used for any application (Jumper et al. 2021; Varadi et al. 2022). The PyMOL Molecular Graphics System, Version 2.0 Schrödinger, LLC, was used to analyze the models and align the best one with 1VNI crystal structure of *Ci*vCPO. The web tool Caver 1.1 was used to calculate the volume of the catalytic pocket of *Hw*vCPO and *Ci*vCPO (Stourac et al. 2019). InterProScan tool was used to analyze the primary structure of *Hw*vCPO (Paysan-Lafosse et al. 2023).

## Results

### The *Hortaea werneckii* Vanadium-dependent Chloroperoxidase Gene Isolation

Following our previous analysis of the fungal genome databases to find fungi which possess genes encoding vanadium haloperoxidases using *Ci*vCPO protein sequence and Blastp tools (Cochereau et al. 2022), one of them from the EXF-2000 genome from *H. werneckii*, attracted our attention as this strain was available in our marine fungal collection. It contained a sequence matching with a vanadium haloperoxidase protein. This strategy was preferred because genomes are available online, along with search tools, thus avoiding a more time-consuming PCR screening and PCR products sequencing. The sequence identified in the EXF-2000 genome from *H. werneckii* contained a sequence matching with the characteristic conserved active site of vHPO, i.e*.*, PxYxSGHA and LxxxxAxxRxxxGxHxxxD (where x represents any amino acid) (Leblanc et al. 2015; Wever et al. 2018) (HWER_13832R0, Protein ID: 8190, Location: scaffold_292:29,540–31,629). Based on this sequence, specific primers were designed to amplify a homologous gene in the isolate *H. werneckii* Mo34 (UBOCC-A-208029) in our culture collection. The nucleotide sequence of the amplified product was obtained and aligned with the sequence of the gene found in *H. werneckii* EXF-2000. The putative protein product corresponded to a 627 amino acid protein sharing 97.2% identity with the putative vHPO from EXF-2000 strain. The alignment with the vCPO sequence of *C. inaequalis* showed that both proteins share 48.41% of identity. In addition, the active site residues are strictly conserved (UniProt entry: P49053). The nucleotide sequence for the *H. werneckii* vHPO is available on GenBank database (*Hw*vCPO, OP555106).

### Phylogenic Study of *Hw*vCPO

**Fig. 2:**
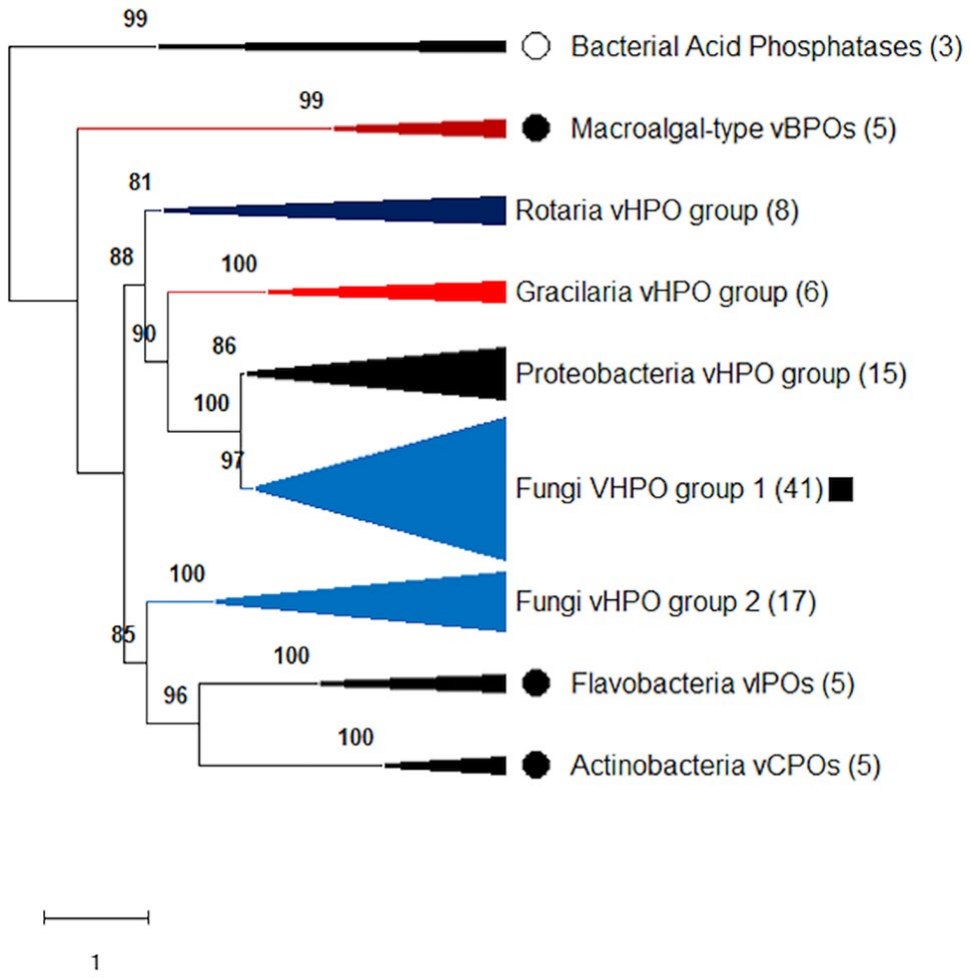
Global phylogenetic analysis showing the positions of the *Hortaea werneckii* and the overall fungal vHPO sequences. The branches including exclusively sequences of bacteria, fungi, rotifers, and rhodophytes are represented respectively in black, blue, dark blue and red colors. The branch composed of a macroalgal and cyanobacterial sequences is shown in brown. Black circles represent the location of biochemically and structurally characterized vHPO enzymes. White circle shows the biochemically and structurally characterized acid phosphatases. Black square identifies the newly discovered vHPO sequence of this study

A phylogenetic study was performed by including sequences from different living organisms from different databases. Among the different sequences used for this phylogenetic study: fungal vHPO sequences are divided into two subgroups: (i) Fungi vHPO group 1 (with 41 sequences) and (ii) Fungi vHPO group 2 (17 sequences) (Fig. 2), and are not clustered together. Fungi vHPO group 1 containing the described vHPO from *H. werneckii* (*Hw*vCPO), *C. inaequalis* (*Ci*vCPO) and *A. didymospora* (*Ad*vCPO) is linked with two other clades: *Rotaria* vHPO group (8 sequences) and *Gracilaria* vHPO group (6 sequences). Fungi vHPO group 1 seems to be more related to Proteobacteria vHPO group as it forms a direct cluster with Proteobacteria sequences supported by a very high bootstrap value (100%). These four monophyletic groups are in a first clade supported by a bootstrap value of 88% (Fig. 2).

The second clade present in this tree is composed of fungi vHPOs group 2 which are more related to Flavobacteria vIPOs (containing vIPOs from *Z. galactanivorans*) and Actinobacteria vCPOs (containing vCPOs from *Streptomyces* sp. CNQ-525). These three monophyletic groups are clustered together with a bootstrap value of 85%. Fungal vHPO in this second group are not described yet (Fig. 2).

There are two other outgroups, composed by bacterial acid phosphatases (3 sequences) and by macroalgal-type vBPOs containing both macroalgal and cyanobacterial proteins (5 sequences). Detailed sequences are available in supplementary information in Table S1.

### Relative Expression of *Hw*vCPO mRNA in 4 Different Culture Media

**Fig. 3:**
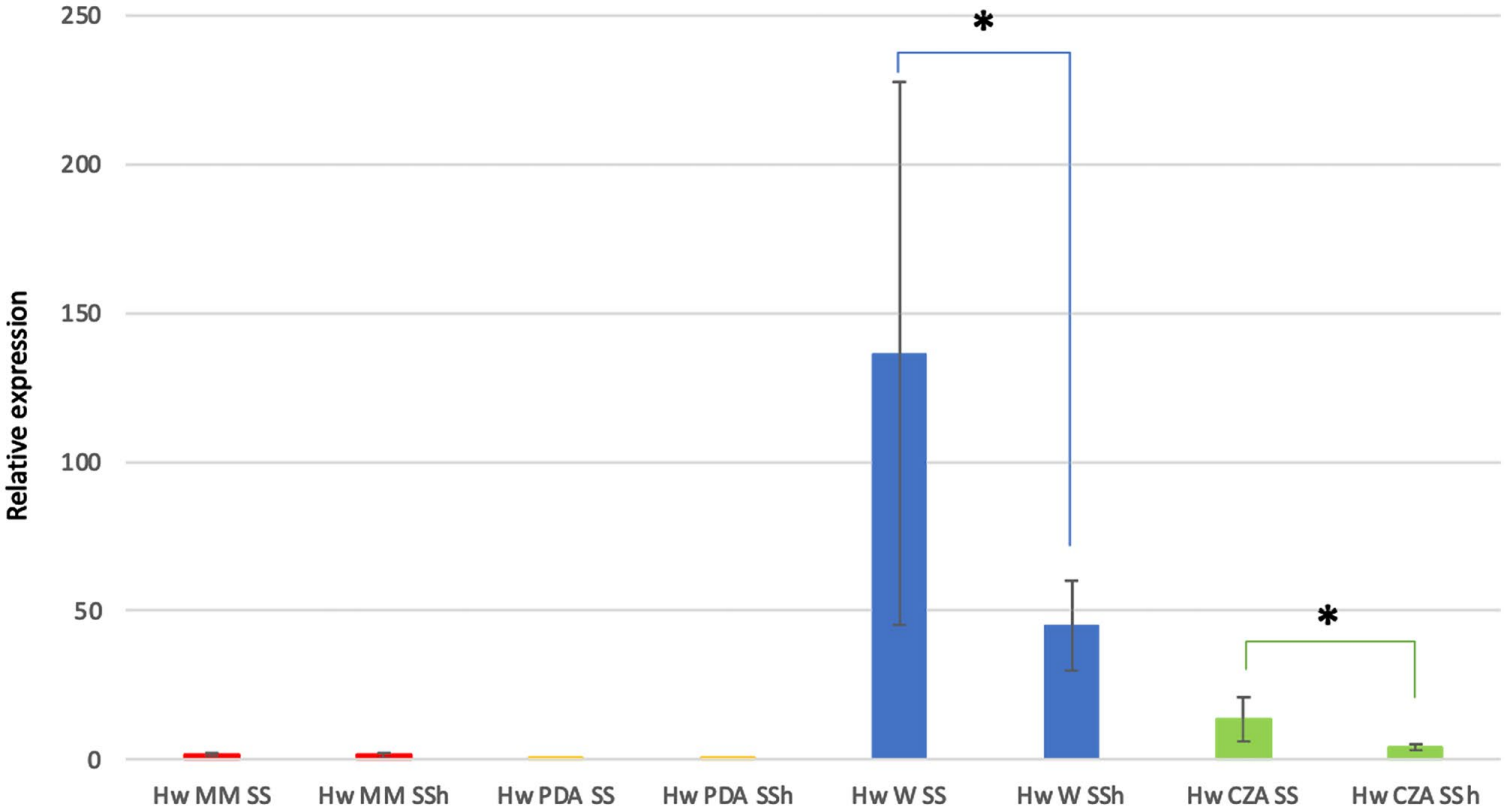
Relative expression of *Hw*vCPO mRNA in different culture media in triplicates (MM: minimal media (red), PDA: potatoes dextrose agar (yellow), W: Wickerham (blue) and CZA: Czapek (green) + SS: sea salts or SSh: sea salts + halogens). Results were calculated using a pre-established standard range of mRNA concentrations (0.5 to 100 ng/µL). Results were normalized using expression of phosphofructokinase mRNA expression. *: Significant difference with addition of halogens in the culture media on the mRNA expression (CI 90)

The nucleotide sequence of the *Hw*vCPO gene made it possible to monitor the expression level of *Hw*vCPO mRNA in *Hortaea werneckii* with 4 different culture media (Fig. 3). The aim here was to compare the mRNA expression between the different media and determine whether the addition of halogens could impact gene expression of this enzyme. Furthermore, we wanted to see if any correlation could be observed between specialized metabolites production by *H. werneckii* and mRNA expression level from the same culture to identify potential target metabolites for in vivo halogenation (HPLC-HRMS analysis, personal data). So far, no significant result has been obtained for halogenated metabolite production.

Our experiments showed that *Hw*vCPO mRNA expression was influenced by the culture conditions. Indeed all the tested conditions show significant difference in mRNA expression (*p*-value < 0.05). mRNA levels were lower in poor culture media such as PDA or Minimal Medium and higher in rich culture media like Wickerham and Czapek. The gene was more highly expressed on the Wickerham culture medium although the variation between “Hw W + SS” replicates was high. Interestingly, for Wickerham and Czapek culture media, supplementation with halogen salts had an impact on the relative mRNA expression. Actually, we detected a significant reduction in SSh media compared to SS media. The halogen concentration in media seems to inhibit *Hw*vCPO mRNA production or affect it stability in these cases. Even if Wickerham medium was identified as the best medium to have the vHPO mRNA expressed, the protein purification in its native form remained unsuccessful, leading to further experiments of heterologous expression.

### Overexpression and Biochemical Characterization of the Recombinant *Hw*vCPO

The gene encoding the *H. werneckii* vanadium chloroperoxidase protein was successfully cloned in the pQE-81L plasmid (Fig. S1). The protein expression in *E. coli* was achieved using a strain harboring the commercial plasmid pKEJ7 to enhance protein refolding (Nishihara et al. 1998, 2000). Without these co-expressed chaperones, vHPO formed inclusion bodies.

The molecular weight of the recombinant protein was approximated at 75 kDa which is consistent with the predicted molecular weight (72.3 kDa). It was validated by a western blot analysis using an anti-His tag antibody (Fig. S2). Surprisingly, the results also showed probable co-purification of chaperone proteins DnaK and DnaJ (protein sizes are consistent with chaperon sizes) with the recombinant vHPO using affinity chromatography. Further purification on gel filtration column was attempted but removal of chaperone proteins was not effective. Indeed, it seemed that chaperones and recombinant vHPO formed a complex or remained linked. The recombinant vHPO was then biochemically characterized using MCD assay, thymol blue assay and *o*-dianisidine assay.

Thymol blue assay was first used to demonstrate iodoperoxidase and bromoperoxidase activities (Verhaeghe et al. 2008) (Fig. S3-S4). This assay successfully validated both iodo- and bromoperoxidase activities of *Hw*vCPO compared to the positive control *Ascophyllum nodosum* vBPO I and diverse negative controls. Indeed, the positive control showed a color change for both iodo- and bromoperoxidase activity which is consistent with a vanadium bromoperoxidase. *Hw*vCPO highlights color changes for both iodine and bromine-containing wells (Fig. S4). Thymol blue assays without vanadate supplementation clearly indicate that *Hw*vCPO is a vanadium-dependent haloperoxidase. Indeed, the addition of vanadate improves the reaction rate of the enzyme obtained with a more intense and faster blue coloration. This indicates a faster formation of brominated thymol blue. Boiled enzymes show no activity during assays validating no artifactual effect driven by medium components (Fig. S3-S4).

**Fig. 4:**
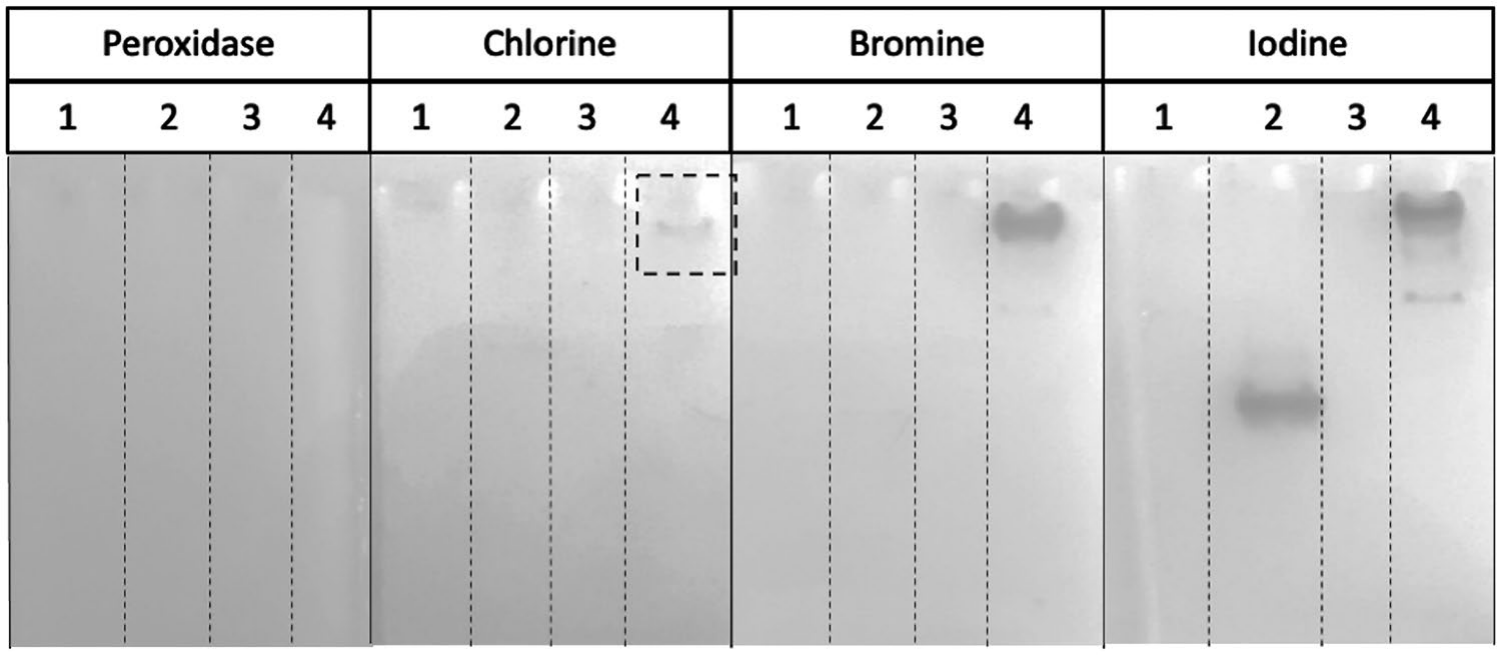
*o*-dianisidine assay presenting the 4 native PAGE conducted on (**1**) the negative control (buffer), (**2**) the positive control *Zg*vIPO1, (**3**) an inactive recombinant vHPO co-expressed with the same chaperon proteins (BL21 + pKJE7 + pQE-80L_vHPO, personal communication), and (**4**) the studied enzyme *Hw*vCPO. Peroxidase, chloroperoxidase, bromoperoxidase, and iodoperoxidase activities were evaluated respectively. Solution 1 contained *o*-dianisidine (100 mM in 1 mL of acetic acid) in 100 mM phosphate buffer pH 7.4 (final volume 100 mL). Solution 2 contained 5 mM H_2_O_2_. First incubation: 50 ml of solution containing 100 mM phosphate buffer pH 7.4, 4.5 mL Solution 1, 1 mM KI, or 2 mM KBr or KCl in ultrapure water, for 1 h with stirring at 20 °C. Second incubation: 50 mL of solution 2 for either 1 h (for KI) or overnight (KCl, KBr and peroxidase) at 20 °C. Peroxidase activity was checked by omitting halogen from the first incubation step (20 °C)

Additionally, haloperoxidase activities were checked on native PAGE using *o*-dianisidine, allowing further investigation of the chloroperoxidase and peroxidase activities (Fig. 4). *Hw*vCPO presents no peroxidase activity but iodo- and bromoperoxidase activities and a weak chloroperoxidase activity. Positive control confirmed previous results based on thymol blue assay as only iodoperoxidase activity was highlighted. Negative control also validated our results since no haloperoxidase or peroxidase activities were revealed. Another assay available in supplementary information validates that the reaction is driven by the presence of the enzyme with a boiled enzyme control showing a loss of activity (Fig. S5).

**Fig. 5:**
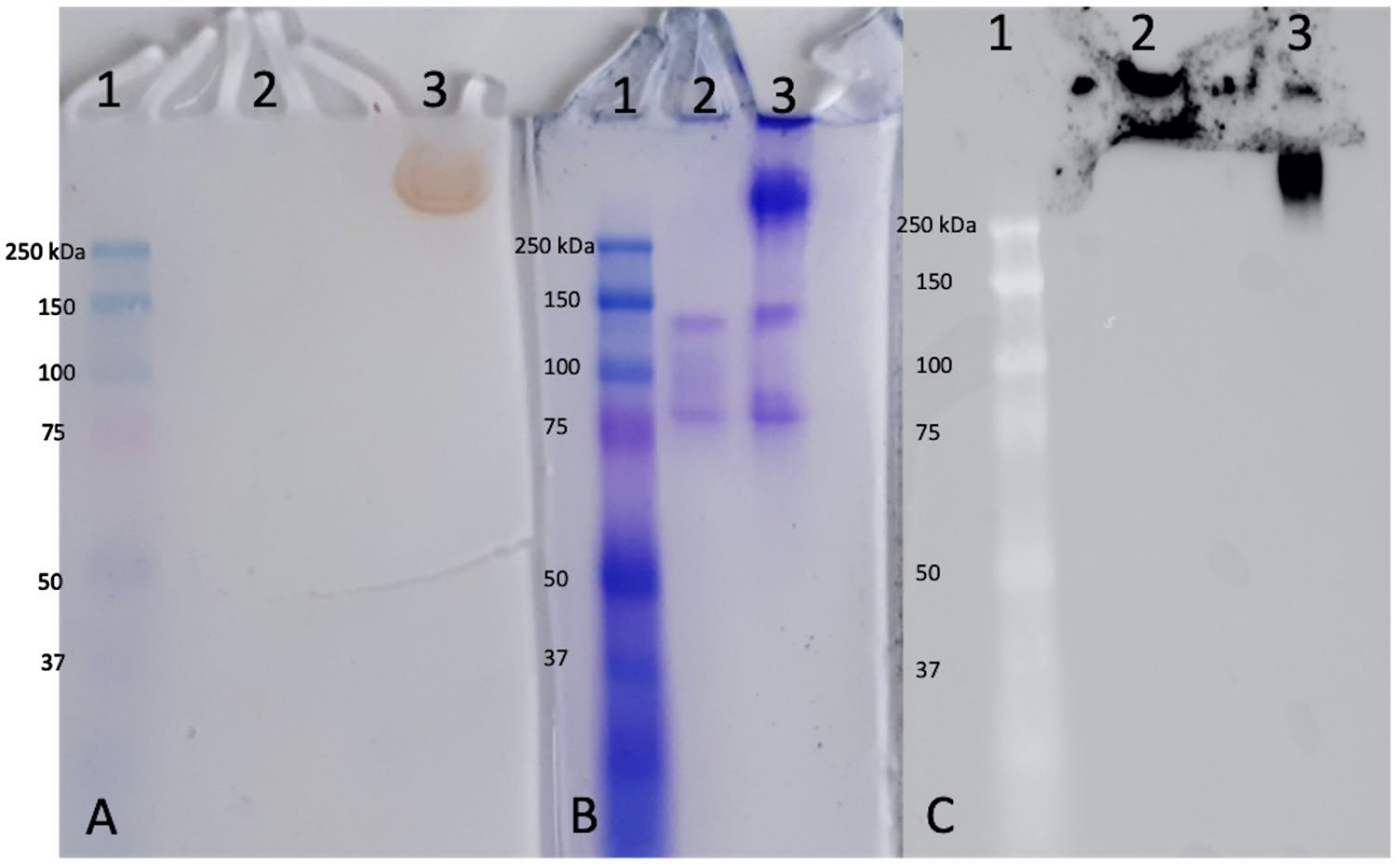
**A** *o*-dianisidine assay to assess bromoperoxidase activity on native PAGE. Lane **1**: size marker (8 µL); Lane **2**: boiled *Hw*vCPO (12 µL); Lane **3**: *Hw*vCPO (12 µL). **B** Native PAGE revealed with Coomassie blue. Lane **1**: size marker (8 µL); Lane **2**: boiled *Hw*vCPO (12 µL); Lane **3**: *Hw*vCPO (12 µL). **C** Western-blot revealed with anti-his tag antibody. Lane **1**: size marker (8 µL); Lane **2**: boiled *Hw*vCPO (12 µL); Lane **3**: *Hw*vCPO (12 µL)

It can be observed on the native PAGE that the native size of HwvCPO is greater than 250 kDa. This observation can be explained by the formation of a complex, possibly with co-expressed chaperone proteins. This specific protein band is also responsible of the enzymatic activity observed during *o*-dianisidine assay (Lane 3 on Fig. 5A). This complex is no longer observable when the enzyme is boiled as well as the corresponding enzymatic activity (Lane 3 on Fig. 5A and lane 2 on Fig. 5B). The thermal denaturation of the enzyme causes its precipitation. Thus, it can no longer penetrate the gel for migration. This explains the signal observed in the stacking for the boiled protein (Lane 2 on Fig. 5C). On the other hand, when the enzyme is not denatured, it can be observed that the complex is indeed composed of the recombinant *Hw*vCPO enzyme because the antibody targets this complex (Lane 3 on Fig. 5C).

**Fig. 6:**
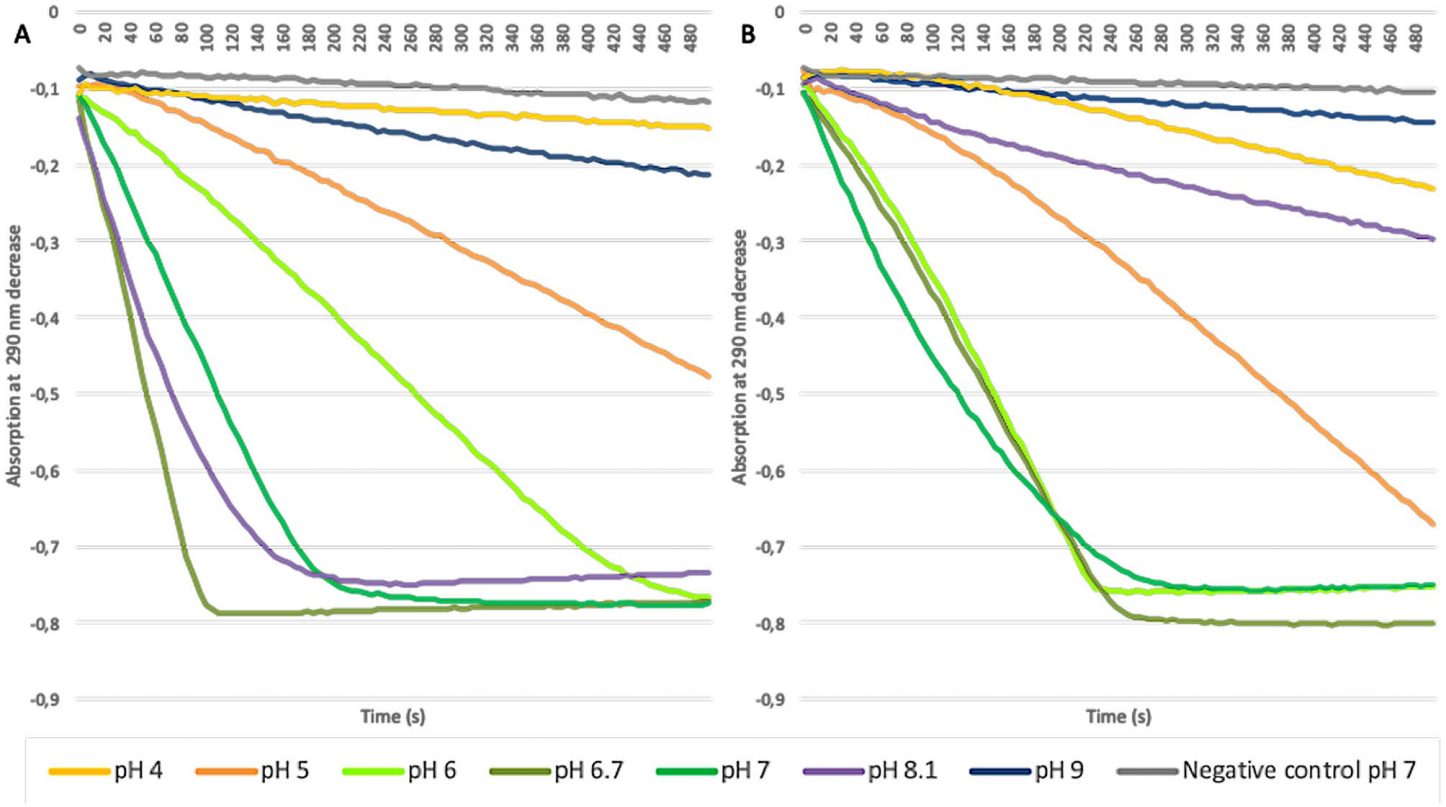
MCD assays with purified recombinant *Hw*vCPO at different pH (pH **4** (yellow), pH **5** (orange), pH **6** (light green), pH **6.7** (khaki green); pH **7** (dark green), pH **8.1** (purple), pH **9** (dark blue)) with both KCl (**A**) and KBr (**B**). The following components were then added to the UV cuvette: 50 mM Buffer (pH **4** to **9**), 50 µM MCD, 200 mM KCl or 200 µM KBr, 50 µL enzymatic solution (5 mg/mL), 10 µM Na_3_VO_4_ and various amounts of distilled water to obtain a final volume of 900 µL. Then 100 µL of H_2_O_2_ 12 mM were added to start the reaction

Finally, by performing a monochlorodimedone (MCD) assay we were able to provide further evidence of chloroperoxidase and bromoperoxidase activities of *Hw*vCPO. The results showed that the optimal pH for *Hw*vCPO activities both with chloride and bromide was pH 6.7 to 8.1 or pH 6 to 7 respectively (Fig. 6A and B).

Following these enzymatic assays, *H. werneckii* vHPO can be described as a vanadium-dependent chloroperoxidase: *Hw*vCPO. Indeed, the enzyme showed activities for both chloride, bromide and iodide with optimal pH around 6–7 (for chlorination and bromination). Higher pH conditions (> 7) led to a drastic decrease in the enzyme activity in the tested conditions for bromide (blue and purple curves, Fig. 6B). While chlorination can still occur at pH 8.1 (purple curve, Fig. 6A), a drastic decrease is also observed for higher pH (blue curve, Fig. 6A). Similarly, chloride and bromide oxidation were not significantly different to negative control levels in acidic conditions (yellow curves, Fig. 6).

### Enzyme Kinetics

**Fig. 7:**
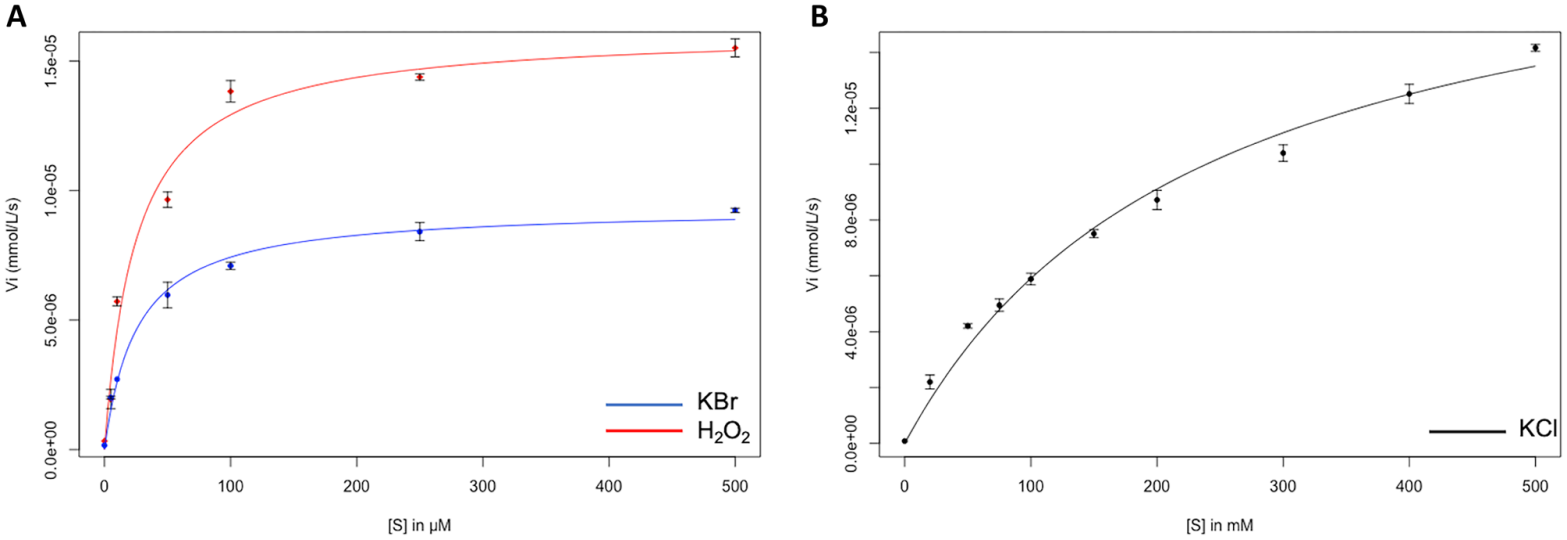
Michaelis–Menten curve obtain for *Hw*vCPO using **A** H_2_O_2_ (red) and KBr (blue) and **B** KCl (black) in MCD assays. MCD assays for KCl and KBr constant calculations were carried as followed: 50 mM buffer pH **7**, 50 µM MCD, 10 µM Na_3_VO_4_, 1.2 mM H_2_O_2_, 1 µL *Hw*vCPO (5 mg/mL), and KCl (20 mM, 50 mM, 75 mM, 100 mM, 150 mM, 200 mM, 300 mM, 400 mM, and 500 mM), KBr (5 µM, 10 µM, 50 µM, 100 µM, 250 µM, and 500 µM). For H_2_O_2_ constant calculations: 100 µM KBr are used and different concentration of H_2_O_2_ (1 µM, 10 µM, 50 µM, 100 µM, 250 µM, and 500 µM). Final volume is 1 mL

Based on Michaelis–Menten curves obtained for the different substrates Cl^−^, Br^−^ and H_2_O_2_ (Fig. 7) with the *Hw*vCPO enzyme at pH 7, *V*_max_, *K*_*m*_, and *k*_cat_ constants for each reaction were calculated (Table 1). Higher concentration of KCl (up to 500 mM) has not been used to avoid enzymatic inhibition (Schijndel et al. 1994).

**Table 1:** Enzymatic parameters obtained by nonlinear regression with Michaelis–Menten equation. These results are the average of three replicates for each substrate (Cl, Br and H_2_O_2_). Standard deviation is indicated in the margin. MCD assays for KCl and KBr constant calculations were carried as followed: 50 mM buffer pH 7, 50 µM MCD, 10 µM Na_3_VO_4_, 1.2 mM H_2_O_2_, 1 µL *Hw*vCPO (5 mg/mL), and KCl (20 mM, 50 mM, 75 mM, 100 mM, 150 mM, 200 mM, 300 mM, 400 mM, and 500 mM), KBr (5 µM, 10 µM, 50 µM, 100 µM, 250 µM, and 500 µM). For H_2_O_2_ constant calculations: 100 µM KBr are used and different concentration of H_2_O_2_ (1 µM, 10 µM, 50 µM, 100 µM, 250 µM, and 500 µM). Final volume is 1 mL

**Substrate**	***V***_**max**_** (µmol/L/s)**	***K***_***m***_** (M)**	***k***_**cat**_** (s**^**−1**^**)**	***k***_**cat**_**/*K***_***m***_** (s**^**−1**^** M**^**−1**^**)**
KCl	0.02 (± 1.59 × 10^**−**4^)	0.237 (± 1.15 × 10^**−**2^)	0.14 (± 1.0 × 10^**−**3^)	5.91 × 10^**−**1^
KBr	0.009 (± 2.18 × 10^**−**4^)	2.63E-05 (± 3.67 × 10^**−**6^)	0.13 (± 9.0 × 10^**−**3^)	4.94 × 10^3^
H_2_O_2_	0.014 (± 1.5 × 10^**−**3^)	1.78E-05 (± 6.55 × 10^**−**6^)	0.22 (± 2.2 × 10^**−**2^)	1.24 × 10^4^

The results showed that *Hw*vCPO possesses downer Michaelis–Menten constant for bromide (*K*_*m*_ = 26 µM) than chloride (*K*_*m*_ = 237 mM) with a 10^4^ factor difference (Table 1). The Michaelis–Menten constant for H_2_O_2_ is in a similar range to bromide with a *K*_*m*_ of 17 µM. The turnover number (*k*_cat_) are in the same range for both chloride and bromide but a little bit higher for hydrogen peroxide. Maximum rates (*V*_max_) are close between the three substrates. For bromide and hydrogen peroxide, catalytic efficiency is respectively 4.94 × 10^3^ and 1.24 × 10^4^ s^−1^ M^−1^, which is below the median catalytic efficiency distribution (1.0 × 10^5^ s^−1^ M^−1^) calculated by Bar-Even et al. (Bar-Even et al. 2011)*.* However, as indicated in this article, our enzyme is among the 60% enzymes that possess a catalytic efficiency between 1.0 × 10^3^ and 1.0 × 10^6^ s^−1^ M^−1^.

### Organic Solvent Tolerance of the Recombinant *Hw*vCPO

**Fig. 8:**
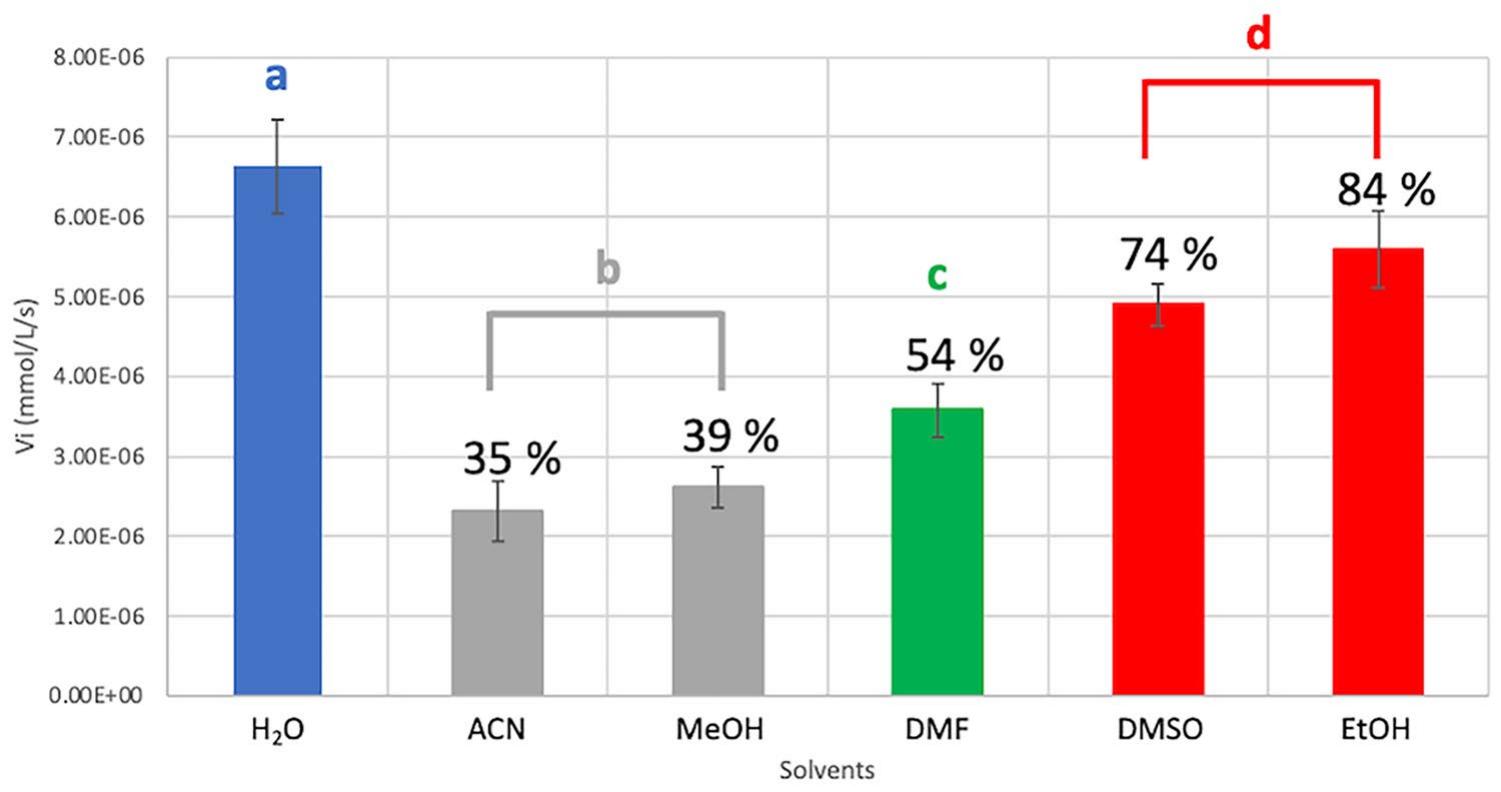
Enzymatic activity of *Hw*vCPO according to the tested solvent (50:50 v/v water:solvent). These assays were carried out in triplicates. H_2_O: water (blue), ACN: acetonitrile (grey group), MeOH: methanol (grey group), DMF: N,N-dimethylformamide (green), DMSO: dimethylsulfoxide and EtOH: ethanol (red group). Groups **a**, **b**, **c** and **d** are discriminated by a one-way ANOVA test (*p*-value of 5.4E-08) coupled to a Fisher LSD test. MCD assays were carried as followed: 50 mM buffer pH 7, 50 µM MCD, 100 µM KBr, 1 µL *Hw*vCPO (5 mg/mL), 10 µM Na_3_VO_4_, 1.2 mM H_2_O_2_ in a 50:50 v/v water/solvent mix to reach a final volume of 1 mL

Considering the potential application of vHPO in biocatalysis, especially regarding their high tolerance to organic solvents (Höfler et al. 2019), the new *Hw*vCPO was evaluated in this context. As many organic molecules are not soluble in distilled water, the possibility of adding organic solvents to the reaction medium widens the potential use of this type of enzyme. The enzymatic activity of *Hw*vCPO in the presence of different organic solvents was then measured. Bartlett test gives a *p*-value of 0.8584, (therefore > 0.05). In fact, the variances can be considered equal and therefore we can carry out an ANOVA. A *p*-value of 5.4E-08 was obtained (< 0.05). This means that there are very highly significant differences between the different conditions (Fig. 8).

Compared to the level of enzymatic activity in distilled water, the closest level of activity was obtained using ethanol and dimethylsulfoxide (respectively 84% and 74% of the enzymatic activity in water). Enzymatic activity in N,N-dimethylformamide decreased by approximatively 50% (54% of the enzymatic activity in water). Acetonitrile and methanol reduced *Hw*vCPO activity more strongly with 35% and 39% of remaining activity compared to the control. Statistical analysis highlights three groups with significant different means: group d gathering ethanol and dimethylsulfoxide tests, group c with only N,N-dimethylformamide and group b with methanol and acetonitrile. These three groups are significantly different from the control test with only water as solvent (group a).

### Structural Analysis Using Bioinformatic Tools

The InterProScan results show that the protein belongs to the superfamily “Phosphatidic acid phosphatase type 2/haloperoxidase (IPR036938)” with a “Phosphatidic acid phosphatase type 2/haloperoxidase (IPR000326)” domain. More precisely, it belongs to the superfamily “Bromoperoxidase/chloroperoxidase C-terminal (IPR016119)” and finally has a domain "Vanadium chloroperoxidase, N-terminal (IPR041067).

**Fig. 9:**
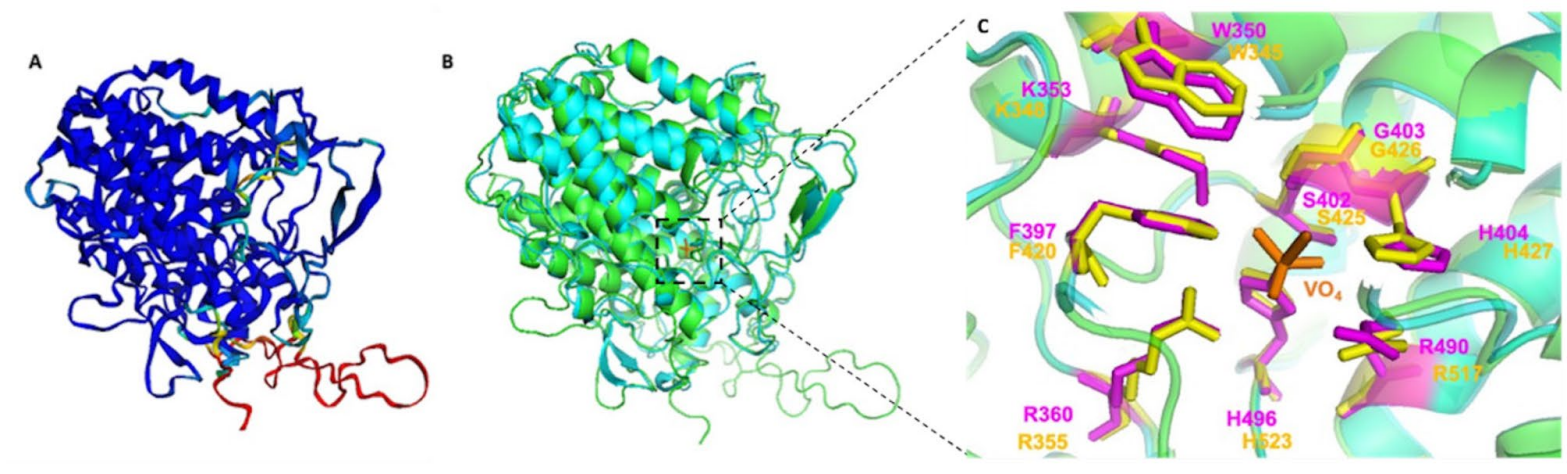
**A** Cartoon 3D structure representation of the best *Hw*vCPO model build using AlphaFold 2. Blue regions indicate good modelling while yellow to red regions indicate uncertain modelling. **B** *Hw*vCPO and *Ci*vCPO secondary structure elements are in green and cyan colored. Vanadate cofactor of the *Ci*vCPO crystallized structure (1VNI) is present in orange at the center of the protein. *Hw*vCPO is represented here by the best model (model 3) structure obtained using AlphaFold2 pipeline analysis. **C** Overlay of the *Ci*vCPO crystallized structure and the best *Hw*vCPO model generated by AlphaFold2. Global structures are showed in cartoon representation where *Hw*vCPO is green and *Ci*vCPO is cyan colored. The 9 residues of the active site are respectively represented in yellow and magenta for *Hw*vCPO and *Ci*vCPO. Vanadate ion is represented in orange

While crystallization of the purified protein was unsuccessful because of the presence of chaperon proteins, its structural analysis was performed using bioinformatics tools. The AlphaFold 2 program was able to produce five models with a high confidence index (88.7 to 89.3 on a 100 scale). The coverage of the sequence with a high pLDDT (predicted Local Distance Difference Test) is important (Mariani et al. 2013). Up to 3000 sequences were found in the databases to align to *Hw*vCPO sequence. Overall, the sequence identity to *Hw*vCPO is low but some sequences share up to 40% sequence identity to query. These sequences were used to build the model. The PAE plots indicate a very similar pattern for all the models that correlates to the pLDDT and sequence coverage per position (supplementary data, Fig. S6). There are 4 regions that exhibit an uncertain probability of folding. These regions are a long insertion loop 363–397 and three shorter zones (35–42, 539–552, and 618–627) and are schematized on the model from yellow to red (Fig. 9A).

This analysis revealed that the structure of *Hw*vCPO is very similar to the structure of *Ci*vCPO, forming a monomeric compact core enzyme composed of 10 alpha helices assembled into 2 distinct bundles. *Ci*vCPO possesses three anti-parallel beta sheets whereas only one of those is present in *Hw*vCPO (an additional study performed with Phyre2 of secondary structures in comparison to CivCPO is available as additional information, Fig. S7). *Ci*vCPO has also an external loop longer than its equivalent in *Hw*vCPO between amino acids 116 and 128 (Fig. 9B). There are five additional loops found at the surface of *Hw*vCPO compared to *Ci*vCPO.

Regarding the active site, *Hw*vCPO exhibits 100% identity of active site amino acids corresponding to the first coordination sphere of vanadate with the 1VNI template (CivCPO, C. inaequalis). This result shows the high conservation of the active site of proteins from the vanadium haloperoxidase family (Fig. 9C)*.* This result is in agreement with the previous alignment of the two sequences.

## Discussion

### Vanadium-Dependent Haloperoxidases in Fungi and Phylogenetic Analysis

The *H. werneckii* vanadium chloroperoxidase described in this work is the fourth to be biochemically characterized in the fungal kingdom. Interestingly, *Hw*vCPO is present in the same monophyletic group as *Ci*vCPO and *Ad*vCPO and it possesses the same halogen specificity. In contrast to algae and marine bacteria where all the vHPO that have been described are bromoperoxidases or iodoperoxidases, fungal vHPO correspond to chloroperoxidases even though their affinity for bromine is higher. To validate the vanadium dependencies of *Hw*vCPO, enzymatic assays were performed without vanadate supplementation after dialysis of pure *Hw*vCPO. It was not possible to completely remove the traces of vanadium present in the purified enzyme and test the apoenzyme. The traces of vanadium certainly originate from the empirical culture media. Therefore, some enzyme activity can be observed when no vanadate supplementation is used, but when vanadate supplementation is performed, better enzyme activity on thymol blue can be observed, suggesting a dependence of *Hw*vCPO for vanadium (Figs. S3 and S4).

The vHPO enzyme family shares a common ancestor with bacterial acid phosphatases as described by Leblanc et al. (Leblanc et al. 2015). However, for the first time, the phylogenetic analysis highlights a common ancestor between proteobacterial vHPOs and fungal vHPOs from the group 1 (Fig. 2). This result is supported by a bootstrap value of 100% which makes it more robust. Therefore, we hypothesize that lateral gene transfer has occurred leading to vHPOs acquisition from proteobacteria to fungi. However, this hypothesis would require further investigation.

Interestingly, a second vHPOs fungal group separated from the first group appears (fungi vHPOs group 2 on Fig. 2). This second group is only composed of putative vHPOs sequences. Many putative vHPOs from Basidiomycetes (*Cyclocybe aegerita*, *Tricholoma matsutake*, *Panaeolus papilionaceus* for example) can be found in this group, which may explain the separation from the group 1 which is only composed of Ascomycetes. Contrary to fungal vHPOs in group 1, these putative vHPOs of group 2 may also have been acquired by horizontal gene transfer from bacteria. The origin of the bacterial lineage involved remains to be elucidated. This discrimination between two vHPOs groups is observed for the first time but sequences from fungi vHPOs group 2 would need to be further characterized to highlight differences between vHPOs from fungi groups 1 and 2 (Fig. 2).

An intraspecific analysis has been performed using the genomic data published by Gostinčar et al. (Gostinčar et al. 2022). This analysis shows that the presence of a gene homologous to *Hw*vCPO is not always the case depending on the *H. werneckii* strain (marine or terrestrial) (Cochereau 2023). The presence of this chloroperoxidase does not therefore seem to be correlated with the high halotolerance of *H. werneckii*. The presence of this protein is not strictly marine as terrestrial strains possess homolog gene in their genome (EXF-151 or EXF-2682 strains).

### Comparing Enzymatic Parameters of *Hw*vCPO with other vHPO

Some trends could be observed while comparing all the vHPOs activities reported in the literature. Specificity constant (*k*_cat_/*K*_*m*_) for bromide was greater compared to chloride (8300 times greater). For *Ci*vCPO, specificity constant for bromide was greater too compared to chloride (950 time greater) (Table 2). Then, this ratio is close to nine times greater for *Hw*vCPO compared to *Ci*vCPO. Moreover, *Hw*vCPO was observed to be more specific for bromide than any vHPOs isolated from red algae, brown algae and bacteria (K_m_ in the mM range for these enzymes). Specificity constant is higher for *A. nodosum* BPO (97 times greater) and *L. digitata* BPO (30 times greater) compared to *Hw*vCPO. Specificity constant for hydrogen peroxide seems to be higher for *Ci*vCPO (469 times greater) compared to *Hw*vCPO (Table 2). Therefore, our work suggests that fungal vHPO are more specific for bromide with a *K*_*m*_ in the µM range and are able to catalyze chlorination in contrast to the algal vHPOs reported here.

**Table 2:** Examples of enzymatically characterized vHPO in all the kingdoms of life

		**K**_**m**_** (mM)**				***k***_**cat**_** (s**^**−1**^**)**				
**Species**	**Clade**	**I**^**−**^	**Br**^**−**^	**Cl**^**−**^	**H**_**2**_**O**_**2**_	**I**^**−**^	**Br**^**−**^	**Cl**^**−**^	**Optimal pH**	**Reference**
*Ascophyllum nodosum* (vBPO II, r)	Brown Algae	NA	0.32	NA	0.022	NA	153	NA	5.9	(Wischang et al. 2012)
*Corallina officinalis* (vBPO, r)	Red Algae	1.80	1.20	NA	0.017	NA	NA	NA	6.5	(Carter et al. 2002)
*Laurencia nipponica* (vBPO 2, r)		NA	0.35	NA	0.025	NA	NA	NA	7.0	(Kaneko et al. 2014)
*Alternaria didymospora* (vCPO, n)	Fungi	NA	0.005	1.2	0.06	NA	60	2	5.3	(Barnett et al. 1998)
*Xanthoria parietina* (vBPO, n)		NA	0.028	NA	0.87	NA	NA	NA	5.5	(Plat et al. 1987)
*Curvularia inaequalis* (vCPO, r)		NA	0.009	0.9	0.035	NA	248	26	5.0	(Hemrika et al. 1999)
*Hortaea werneckii* (vCPO, r)		NA	0.026	237	0.017	NA	0.13	0.14	6–8	This work
*Streptomyces* sp. *CNQ-525* (NapH1, r)	Bacteria	NA	NA	4	0.001	NA	NA	NA	6.0	(Bernhardt et al. 2011)
*Acaryochloris marina* (vBPO, r)	Cyano-bacteria	6.40	0.40	NA	0.06	NA	8.40	NA	6.0	(Frank et al. 2016)
*Synechococcus* sp. *CC9311* (vBPO, n)		0.02	1.50	NA	NA	NA	NA	NA	7.0	(Johnson et al. 2011)

*NA* not available, *r* recombinant protein, *n* native protein

Contrary to other vHPOs presented in Table 2, *Hw*vCPO possess a weak turnover number (*k*_cat_). This constant may be impacted by the presence of chaperones which can form a complex with *Hw*vCPO. It would be interesting to calculate the turnover number for a range of pH from 6 to 8.1 to optimize the *k*_*c*at_ value. This lower value could also be the consequence of the closing of the 2nd active site entry and the deeper active site pocket in *Hw*vCPO predicted by the structural analysis of the AlphaFold2 model. In this case, fewer halogens may enter the active site and the longer distance to reach the vanadate cofactor could result in a reduction in the maximum number of chemical conversions of substrate molecules per second.

Any previously described vCPO seem to possess better affinity for bromide compared to chloride. The higher specificity for bromination over chlorination in fungi (even for terrestrial-derived strains) might be explained by the scarcity of bromine compared to chlorine in both terrestrial and marine environments. Indeed, higher specificity for bromide can be explained because bromine is rarer than chlorine in both terrestrial ([Cl]: 320 mg/kg; [Br]: 3 mg/kg) and marine environments ([Cl]: 19,000 mg/kg; [Br]: 65 mg/kg) (Gribble 2010). The *Ci*vCPO from terrestrial origin seems to be more impacted by pH changes compared to *Hw*vCPO. Indeed, the affinity of *Ci*vCPO for bromide at pH 7 remains in the same range but the catalytic constant is significantly reduced (*K*_*m*_: 10 µM but the *k*_cat_ decreases by up to 40 times from 248 to 6 s^−1^). *Ci*vCPO has optimal activity at acidic pH ranging from 4.2 to 6.3 (Hemrika et al. 1999). Contrary to CivCPO, *Hw*vCPO described here, has an optimal pH value in a neutral pH range (pH 6–8.1, Fig. 6).

Some limitations in the comparison of enzymatic activities can be here discussed and acknowledged. Strict comparison appears difficult since the enzymatic activity of *Hw*vCPO was purified as a recombinant protein from *E. coli*, potentially impacting its activity, while the other described vHPOs have been directly purified from their natural producers. For example, chaperone proteins were used to enhance *Hw*vCPO folding and these proteins remained linked to the recombinant *Hw*vCPO and were still detected after purification. Therefore, we cannot exclude the possibility that these chaperone proteins might skew the enzymatic activity measured. It would thus be interesting in future investigations (i) to compare this recombinant enzyme with the native *Hw*vCPO purified directly from *H. werneckii*, and/or (ii) to overexpress the other described vHPOs using the same approach as for *H. werneckii*.

### *Hw*vCPO Organic Solvent Tolerance

According to the results presented in Fig. 8, *Hw*vCPO remains active in different organic solvents without losing too much activity comparing to its activity in water DMF and DMSO tolerance appear interesting for further investigations because these solvents are frequently used in fine chemical industries (Roy 2000; Lynch 2003). The wide range solvent tolerance observed is also of interest in terms of potential applications in biocatalysis because it allows the solubilization of different compounds which cannot be dissolved in water. Early assays were carried out using H_2_O/ACN (50:50 v/v) or H_2_O/EtOH (50:50 v/v) and enzymatic bromination, di-bromination, or tri-bromination of different compounds (thymol, phloroglucinol) were successfully obtained (data not shown).

### Structural Analysis of the AlphaFold2 Model Obtained for *Hw*vCPO

**Fig. 10:**
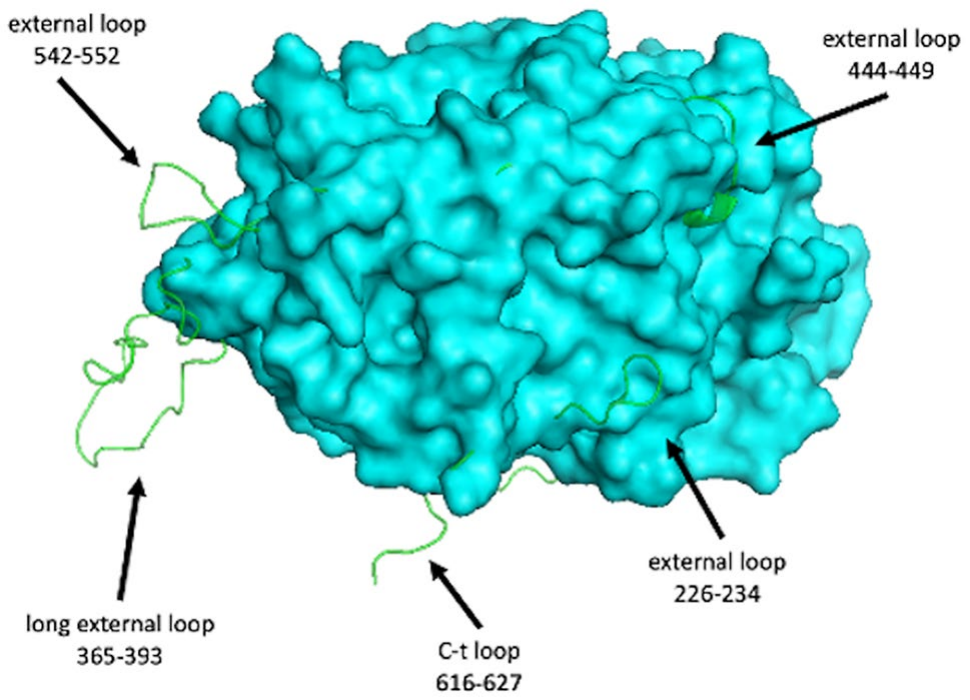
The overall 3D structure of *Ci*vCPO is represented by a surface view (cyan color). The 4 additional loops of *Hw*vCPO (green color) are modelled by overlay showing external extensions outside the *Ci*vCPO structure

**Fig. 11:**
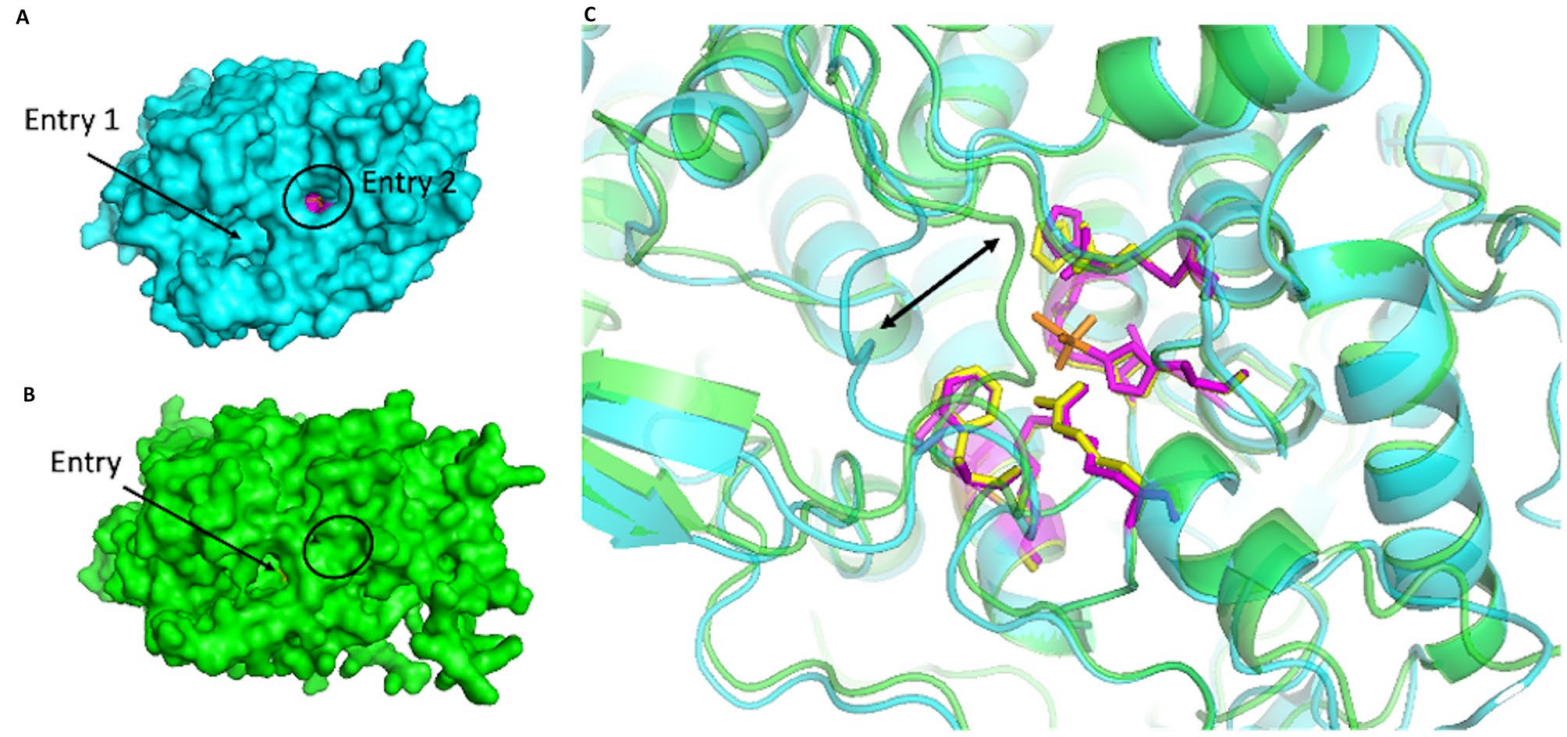
Global view of the second active site entry of *Ci*vCPO (**A**) masked in *Hw*vCPO (**B**). (**C)** Global structures are showed in cartoon representation where *Hw*vCPO is green and *Ci*vCPO is cyan colored. The 9 residues of the active site are respectively represented in yellow and magenta for *Hw*vCPO and *Ci*vCPO. Vanadate ion is represented in orange. The shift zone is indicated by a black double arrow

Based on our predicted model, new structural elements were found in the *Hw*vCPO enzyme compared to *Ci*vCPO, notably five additional loops at the surface of the enzyme. Four of these loops are localized far from the active site entrance (Figs. 10 and 11). The potential role(s) of these supplementary domains, particularly the longer external loop 365–393 (Fig. 10), remains to be determined.

Another difference between the two proteins concerns the accessibility of the active site of the enzyme. In *Ci*vCPO, there are 2 potential active site gate entries for the halogen ion while in *Hw*vCPO, only one entry leads to the vanadate cofactor (Fig. 11A and B). This difference can be explained by a modification in a loop close to the active site. The equivalent loop 389–396 of *Ci*vCPO in *Hw*vCPO exhibits a shift that triggers the entire covering of the entrance site 2. The “NRIPFKPA” sequence present in the *Hw*vCPO loop is replaced by “NDIPFKPP” in *Ci*vCPO and it contains 2 prolines instead of 3 in the second case. Consequently, it modifies in *Hw*vCPO the angle of the loop inclination that results in entrance obstruction that provokes the entry closing (Fig. 11C).

**Fig. 12:**
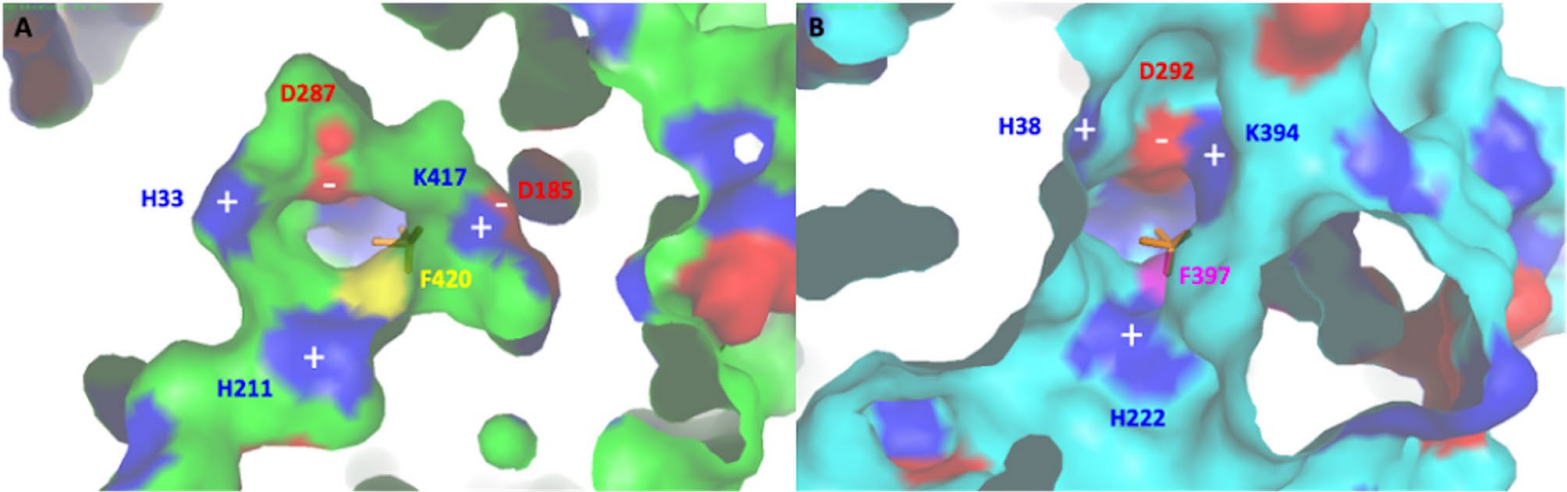
Comparison of the active site entries of *Hw*vCPO (**A**) and *Ci*vCPO (**B**), Vanadate cofactor, added by overlay of *Ci*vCPO structure (1VNI), is colored in orange and visible by transparency in the catalytic pocket. Deep view of the enzyme is represented by surface appearance of the amino acids. Basic and acidic amino acid residues are respectively colored in blue and red. Neutral residues are in green (**A**) and cyan (**B**). Phenylalanine residue suspected to be the acceptor amino acid for binding of X^−^ (Cl^−^ or Br.^−^) is colored in yellow (F420, **A**) or magenta (F397, **B**) (Messerschmidt and Wever 1996)

Further analysis of the electrostatic charge around the entry site revealed that three basic (H33, H211, and K417) and one acid (D287) residues are found at the surface in the most internal active site entrance. An equivalent distribution and nature of charged residues (H38, H222, K394, and D292) are present in *Ci*vCPO (Fig. 12). Nevertheless, K417 is part of the shifted loop in *Hw*vCPO resulting in a more distant position of this amino acid from the entrance compared to *Ci*vCPO (K394, Fig. 12). D185 is near K417 and could also reduce the global electrostatic charge around the cavity entry in *Hw*vCPO. This difference could also affect the halogen specificity of the two enzymes (Fig. 12). The electrostatic charge of the second entrance of *Ci*vCPO, is shown below: three positive charges (R360, K394, and R490) and one negative charge (D292). Thus, in each case, the electrostatic charge of the entry site is favorable for halide attraction. The difference in the number of site entries between the two enzymes may explain some differences observed in the substrate selection and affinity.

The global disposition and number of charges around the surface near the active site access are also slightly different between the 2 enzymes. The *Hw*vCPO entry site is surrounded by potential positive charges mediated by 6 basic amino acids and the potential negative charges of 6 acidic residues form a second circle around the positive charges. In *Ci*vCPO, there is also a potential positive halo around the active site entry composed of 6 basic acidic amino acids, but it appears to cover a more extended surface and includes one central negative charge absent in *Hw*vCPO. This may be the consequence of the presence of two entries to the active site in *Ci*vCPO (Supplementary data, Fig. S8).

**Fig. 13:**
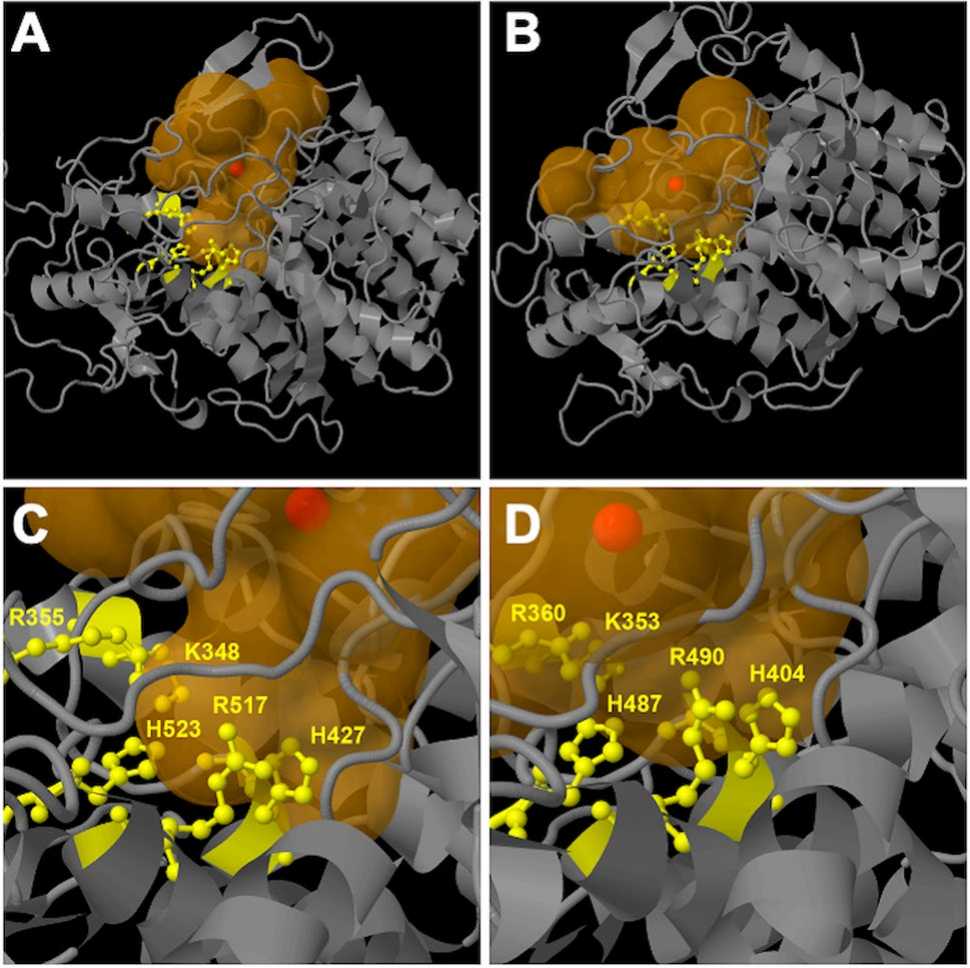
Representation of the catalytic pocket cavities predicted by Caver v1.2 for the overall *Hw*vCPO (**A**) and *Ci*vCPO (**B**) enzymes. Zoom of the catalytic residues regions for *Hw*vCPO (**C**) and *Ci*vCPO (**D**). Protein backbones are shown in grey and cavities are colored in light brown. Catalytic amino acids are in yellow and the cavity centers are shown as red balls

The number of site entries is not the only difference between the two active site pockets. Indeed, in all the models predicted by AlphaFold 2, the catalytic pocket volume is more important in *Hw*vCPO (mean for model 3: 2295 Å^3^) comparing to *Ci*vCPO (mean: 1774 Å^3^) (supplementary data, Table S2). The overall shape of the pocket for *Hw*vCPO is more elongated compared to the pocket of *Ci*vCPO and its catalytic part containing vanadate and key histidine residues is located deeper inside the core of the protein. Therefore, the halogen atom therefore has more distance to cover to reach the vanadate cofactor (Fig. 13).

As previously discussed, the first sphere of coordination around the vanadate cofactor is 100% identical between *Hw*vCPO and *Ci*vCPO. Fine displacements can induce some modifications in affinity and/or catalytic constants.

In summary, structural analysis revealed some differences between *Hw*vCPO and *Ci*vCPO in the number of entrances toward to the active site and the electrostatic potential surrounding the entrance. The residues positioned at the entrance itself allow the same electrostatic potential to attract halogen ions. However, in *Hw*vCPO the partial positive charge seems to be lower compared to *Ci*vCPO because K417 is more distant from the entrance. In addition, the catalytic pocket of the two proteins seems different. The *Hw*vCPO catalytic pocket volume is greater than *Ci*vCPO and the catalytic residues are found deeper in the model of *Hw*vCPO.

## Conclusion

We have overexpressed and biochemically described a new vanadium-dependent chloroperoxidase from the deep-sea marine fungus *H. werneckii* UBOCC-A-208029. This enzyme was described as a vanadium-dependent chloroperoxidase which can oxidize chloride, bromide, and iodide. This protein is being investigated for the development of greener halogenating chemistry tools and has potentially broader applications based on its activity in the presence of different organic solvents. This characteristic is useful for halogenating a wide range of hydrophobic substrates. In terms of perspective, future analyses, including the crystallization of the recombinant protein and the production of enzyme mutants will be conducted to better understand the role of associated chaperones in the macromolecular structure and the molecular bases of halogen specificity of *Hw*vCPO. Moreover, the implication of *Hw*vCPO in halogenated metabolite production has not been depicted and the in vivo function of this enzyme remains to be elucidated.

## Supplementary Information

Below is the link to the electronic supplementary material.

Supplementary file1 (DOCX 8726 KB)

Supplementary file2 (DOCX 8726 KB)

## Data Availability

The datasets used in the present study are available from the corresponding author on reasonable request. *Hortaea werneckii* (UBOCC-208029) strain is available in the UBO Culture Collection (https://www.univ-brest.fr/ubocc/fr). The nucleotide sequence for the *H. werneckii* vCPO is available on GenBank database (*Hw*vCPO, OP555106).
