# Supplementary material for: Heterologous Expression and Biochemical Characterization of a New Chloroperoxidase Isolated from the Deep-Sea Hydrothermal Vent Black Yeast Hortaea werneckii UBOCC-A-208029
Source: Mar Biotechnol (NY). 2023 Jun 24;25(4):519–36. doi: 10.1007/s10126-023-10222-7 (PMC10427571; doi:10.1007/s10126-023-10222-7)
Supplement: Supplementary file 1 — Supplementary file1 (DOCX 8726 KB) [file 10126_2023_10222_MOESM1_ESM.docx]

Supplementary Material

## Supplementary Figures

Fig. S1. Map of pQE-81L_*Hw*vCPO used to overexpress the recombinant protein. *Hw*vCPO gene is in orange. Start codon is identified as “ATG” and the “6xHis” tag is located in the N-terminal part of the recombinant protein.

Fig. S2. (A) 12% SDS-PAGE of purified recombinant vHPO. (B) Western-Blot with same samples. Lane 1: size marker, lane 2: crude extract BL21 + pKJE7 + pQE81L_*Hw*vCPO; lane 3: empty well (some overflow from well 2 is observed); lane 4: purification Ni-NTA fraction 10; lane 5: purification Ni-NTA fraction 11; lane 6: purification Ni-NTA fraction 12; lane 7: purification Ni-NTA fraction 13; lane 8: purification Ni-NTA fraction 14; lane 9: purification Ni-NTA fraction pooled; lane 10: Gel filtration fraction 19; lane 11: Gel filtration fraction 20; lane 12: Gel filtration fraction 21; lane 13: Gel filtration F19 to F21 concentration (some overflow from well 14 is observed); lane 14: size marker.


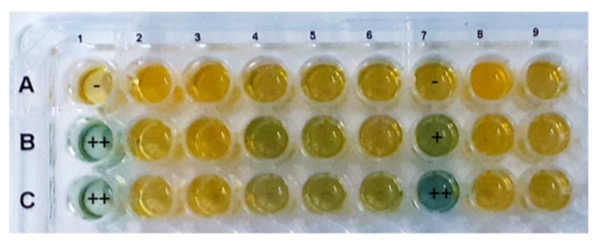


Fig. S3. Thymol blue assay to assess bromoperoxidase activity after 30 minutes of reaction at 20°C. Raw A: boiled enzymes; Raw B: thymol blue assay without vanadate supplementation; Raw C: thymol blue assay with vanadate supplementation. Lane 1: positive control *An*vBPO I (500 ng); Lane 2: crude extract BL21 + pKJE7+ pQE-81L_*Hw*vCPO (5 µL); Lane 3: BL21 + pKJE7+ pQE-81L_*Hw*vCPO unbound proteins after purification on Ni-NTA column (5 µL); Lane 4: purified *Hw*vCPO fraction 12 after Ni-NTA purification (5 µL); Lane 5: pool of purified *Hw*vCPO before gel filtration (5 µL); Lane 6: *Hw*vCPO fraction 20 after Gel filtration purification (5 µL); Lane 7: concentrated *Hw*vCPO after Gel filtration purification (2.5 µg); Lane 8: crude extract negative control BL21 + pKJE7 (5 µL); Lane 9: negative control buffer used during gel filtration (5 µL).

Fig. S4. Thymol blue assay to assess bromoperoxidase and iodoperoxidase activity at 20°C. Row A: boiled enzymes; Row B: thymol blue assay with vanadate supplementation; Row C: thymol blue assay without vanadate supplementation. Lane 1: negative control buffer used during gel filtration (5 µL); Lane 2: positive control *An*vBPO I (500 ng); Lane 3: concentrated *Hw*vCPO after Gel filtration purification (2.5 µg).

Fig. S5. (A) *o*-dianisidine assay to assess bromoperoxidase activity. Lane 1: boiled positive control *An*vBPO I (10 µL); Lane 2: boiled concentrated *Hw*vCPO after Gel filtration purification without vanadate supplementation (30 µL); Lane 3: boiled concentrated *Hw*vCPO after Gel filtration purification with vanadate supplementation (30 µL); Lane 4: size marker (10 µL); Lane 5: positive control *An*vBPO I (10 µL); Lane 6: concentrated *Hw*vCPO after Gel filtration purification without vanadate supplementation (30 µL); Lane 7: concentrated *Hw*vCPO after Gel filtration purification with vanadate supplementation (30 µL). (B) native protein gel colored with Coomassie blue; Lane 1: boiled positive control *An*vBPO I (5 µL); Lane 2: boiled concentrated *Hw*vCPO after Gel filtration purification without vanadate supplementation (15 µL); Lane 3: boiled concentrated *Hw*vCPO after Gel filtration purification with vanadate supplementation (15 µL); Lane 4: size marker (10 µL); Lane 5: positive control *An*vBPO I (5 µL); Lane 6: concentrated *Hw*vCPO after Gel filtration purification without vanadate supplementation (15 µL); Lane 7: concentrated *Hw*vCPO after Gel filtration purification with vanadate supplementation (15 µL).


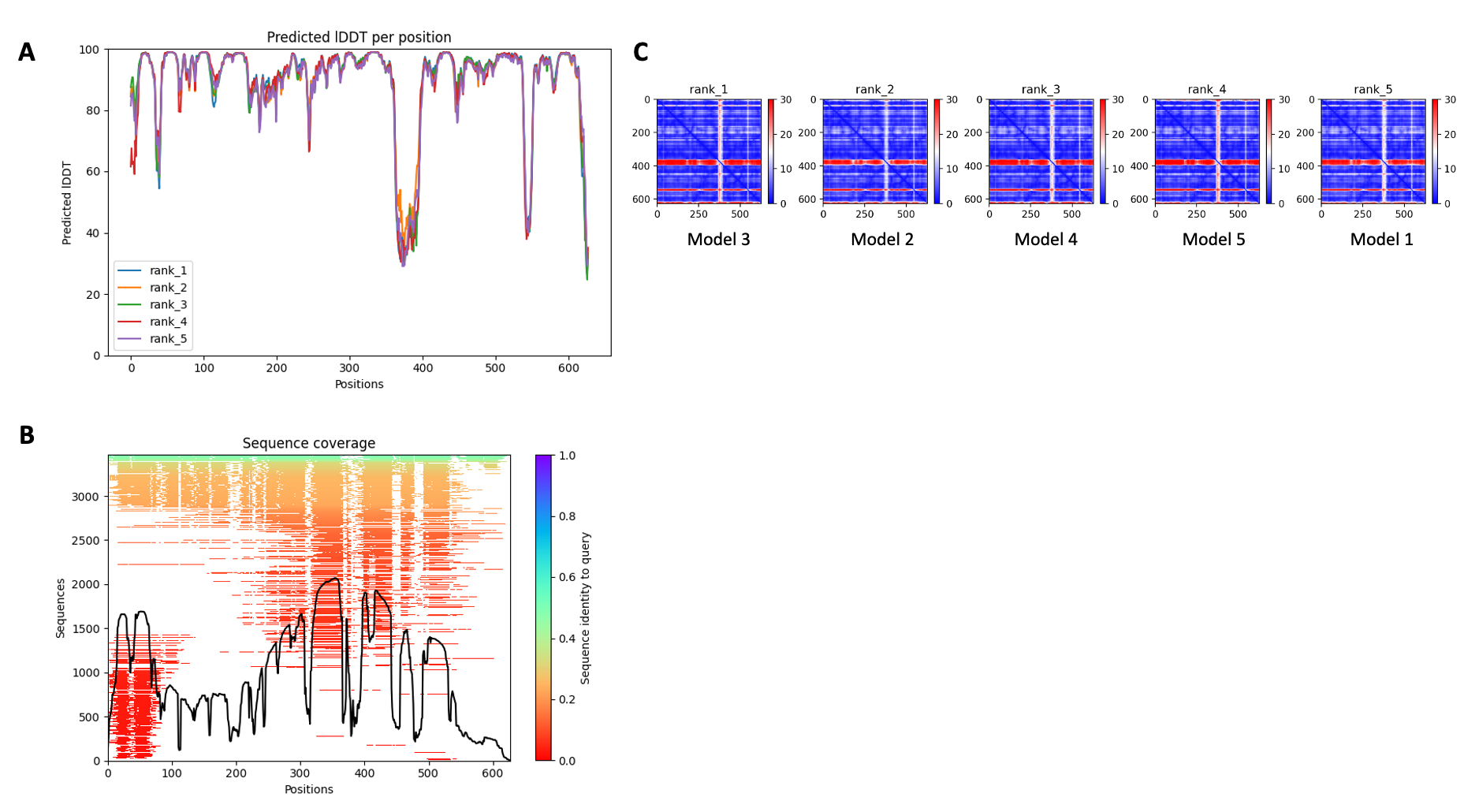


**Fig. S6.** (A) Analysis of the quality confidence of the 5 *Hw*vCPO models pLDDT per position. (B) Analysis of sequence coverage of *Hw*vCPO per position. (C) Plots of the predicted alignment error (PAE) identified per *Hw*vCPO model.


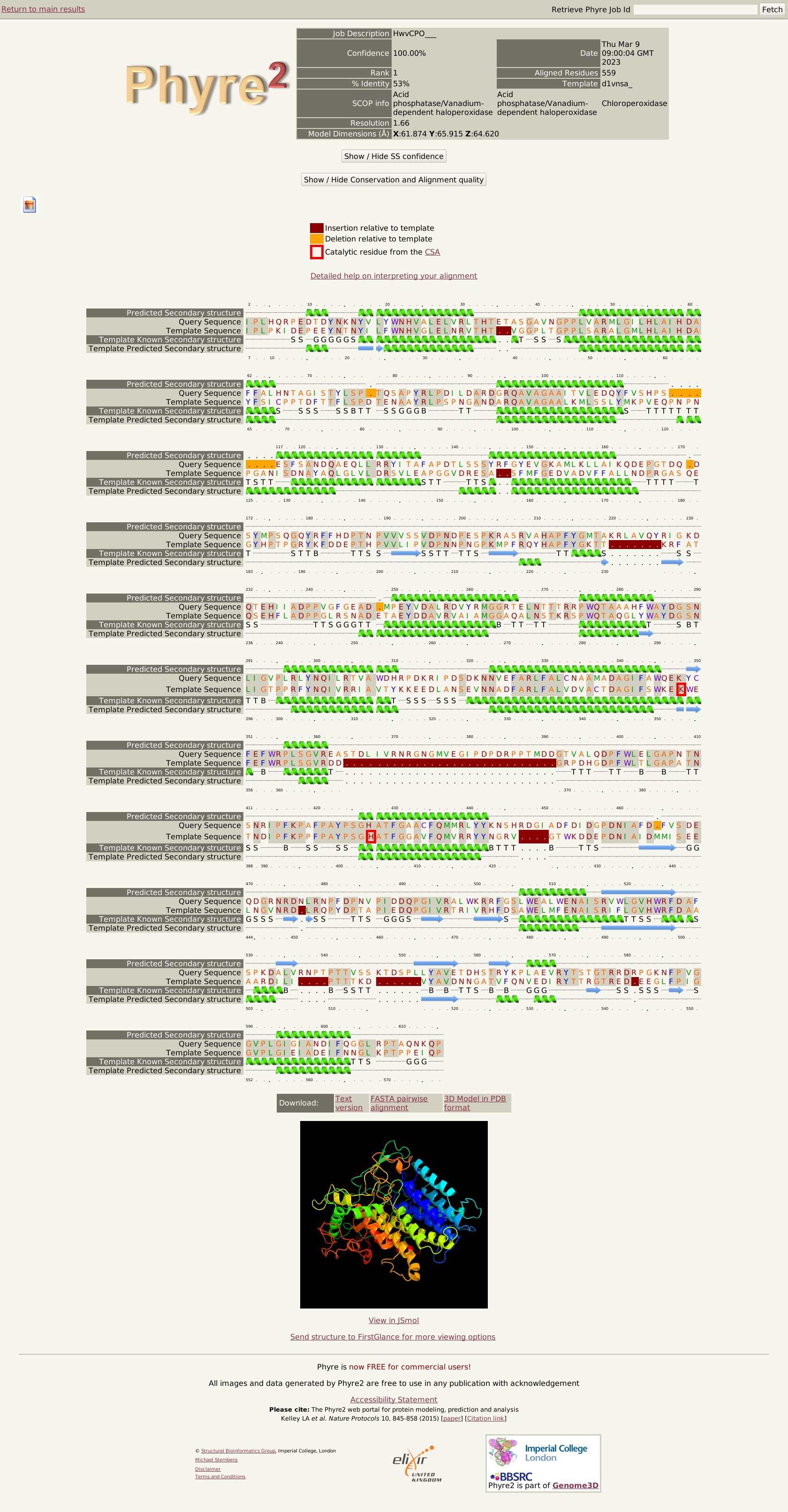


**Fig. S7.** Phyre2 alignment analysis of *Hw*vCPO sequence (query) and the template sequence of CivCPO (PDB ID: 1VNS).

Fig. S8. Potential charges surrounding active site entries for (A) *Hw*vCPO and (B) *Ci*vCPO.

**Table S1.** Proteins sequences used to build the phylogenetic tree.

| **Accession** | **Specie/strain** | **Phylum** | **Sequence group** |
| --- | --- | --- | --- |
| P26976.1 | Salmonella enterica | Proteobacteria | Bacterial acid phosphatases |
| 1D2T | Shimwellia blattae | Proteobacteria | Bacterial acid phosphatases |
| BAA33148.1 | Prevotella intermedia | Bacteroidetes | Bacterial acid phosphatases |
| 5AA6 | Ascophyllum nodosum | Phaeophytes | Macroalgal-type vBPOs |
| 1QI9 | Ascophyllum nodosum | Phaeophytes | Macroalgal-type vBPOs |
| 5LPC | Acaryochloris marina | Cyanobacteria | Macroalgal-type vBPOs |
| 1QHB | Corallina officinalis | Rhodophytes | Macroalgal-type vBPOs |
| 1UP8 | Corallina pilulifera | Rhodophytes | Macroalgal-type vBPOs |
| CAF1181624.1 | Didymodactylos carnosus | Rotifers | Rotaria vHPO group |
| CAF1614509.1 | Adineta ricciae | Rotifers | Rotaria vHPO group |
| UJR11683.1 | Adineta vaga | Rotifers | Rotaria vHPO group |
| CAF1514839.1 | Rotaria sordida | Rotifers | Rotaria vHPO group |
| CAF1548122.1 | Rotaria magnacalcarata | Rotifers | Rotaria vHPO group |
| CAF2530422.1 | Rotaria sp. | Rotifers | Rotaria vHPO group |
| CAF3411370.1 | Rotaria socialis | Rotifers | Rotaria vHPO group |
| CAF4637066.1 | Rotaria sp. | Rotifers | Rotaria vHPO group |
| KAI0558688.1 | Gracilaria domingensis | Rhodophytes | Gracilaria vHPO group |
| PXF42802.1 | Gracilariopsis chorda | Rhodophytes | Gracilaria vHPO group |
| PXF44366.1 | Gracilariopsis chorda | Rhodophytes | Gracilaria vHPO group |
| PXF44371.1 | Gracilariopsis chorda | Rhodophytes | Gracilaria vHPO group |
| PXF44372.1 | Gracilariopsis chorda | Rhodophytes | Gracilaria vHPO group |
| PXF49503.1 | Gracilariopsis chorda | Rhodophytes | Gracilaria vHPO group |
| WP_194842858.1 | Agrobacterium sp. | Proteobacteria | Proteobacteria vHPO group |
| WP_203020174.1 | Rhizobium rosettiformans | Proteobacteria | Proteobacteria vHPO group |
| WP_207458222.1 | Azospirillum sp. | Proteobacteria | Proteobacteria vHPO group |
| WP_056624028.1 | Sphingomonas sp. | Proteobacteria | Proteobacteria vHPO group |
| WP_082451463.1 | Sphingomonas sp. | Proteobacteria | Proteobacteria vHPO group |
| WP_126974601.1 | Frigidibacter oleivorans | Proteobacteria | Proteobacteria vHPO group |
| WP_131578347.1 | Paracoccus nototheniae | Proteobacteria | Proteobacteria vHPO group |
| WP_147829302.1 | Methylobacterium sp. | Proteobacteria | Proteobacteria vHPO group |
| WP_165214374.1 | Rhizobium pseudoryzae | Proteobacteria | Proteobacteria vHPO group |
| WP_167623604.1 | Paracoccus sp. | Proteobacteria | Proteobacteria vHPO group |
| WP_168217335.1 | Paracoccus liaowanqingii | Proteobacteria | Proteobacteria vHPO group |
| WP_170149750.1 | Rhodoplanes roseus | Proteobacteria | Proteobacteria vHPO group |
| WP_171901632.1 | Agrobacterium sp. | Proteobacteria | Proteobacteria vHPO group |
| WP_222060999.1 | Rhizobium laguerreae | Proteobacteria | Proteobacteria vHPO group |
| WP_252574241.1 | Sphingomonas aerolata | Proteobacteria | Proteobacteria vHPO group |
| XP_043130311.1 | Aspergillus viridinutans | Ascomycetes | Fungi VHPO group 1 |
| XP_007780731.1 | Coniosporium apollinis | Ascomycetes | Fungi VHPO group 1 |
| XP_007784037.1 | Coniosporium apollinis | Ascomycetes | Fungi VHPO group 1 |
| XP_018032291.1 | Paraphaeosphaeria sporulosa | Ascomycetes | Fungi VHPO group 1 |
| XP_008025383.1 | Exserohilum turcica | Ascomycetes | Fungi VHPO group 1 |
| CAA59686.1 | Curvularia inaequalis | Ascomycetes | Fungi VHPO group 1 |
| XP_007691748.1 | Bipolaris oryzae | Ascomycetes | Fungi VHPO group 1 |
| XP_007694919.1 | Bipolaris sorokiniana | Ascomycetes | Fungi VHPO group 1 |
| XP_007711916.1 | Bipolaris zeicola | Ascomycetes | Fungi VHPO group 1 |
| XP_014084379.1 | Bipolaris maydis | Ascomycetes | Fungi VHPO group 1 |
| XP_014562472.1 | Bipolaris victoriae | Ascomycetes | Fungi VHPO group 1 |
| CAA72622.1 | Alternaria didymospora | Ascomycetes | Fungi VHPO group 1 |
| XP_028505902.1 | Alternaria arborescens | Ascomycetes | Fungi VHPO group 1 |
| XP_043169711.1 | Alternaria atra | Ascomycetes | Fungi VHPO group 1 |
| XP_046031909.1 | Alternaria rosae | Ascomycetes | Fungi VHPO group 1 |
| XP_038790776.1 | Alternaria burnsii | Ascomycetes | Fungi VHPO group 1 |
| XP_018385764.1 | Alternaria alternata | Ascomycetes | Fungi VHPO group 1 |
| XP_001933850.1 | Pyrenophora tritici-repentis | Ascomycetes | Fungi VHPO group 1 |
| XP_033551421.1 | Lindgomyces ingoldianus | Ascomycetes | Fungi VHPO group 1 |
| XP_033684351.1 | Trematosphaeria pertusa | Ascomycetes | Fungi VHPO group 1 |
| XP_008079410.1 | Glarea lozoyensis | Ascomycetes | Fungi VHPO group 1 |
| XP_037150589.1 | Letharia lupina | Ascomycetes | Fungi VHPO group 1 |
| XP_037158438.1 | Letharia columbiana | Ascomycetes | Fungi VHPO group 1 |
| HwvCPO | Hortaea werneckii UBOCC-A-208029 | Ascomycetes | Fungi VHPO group 1 |
| RMX79094.1 | Hortaea werneckii EXF-6656 | Ascomycetes | Fungi VHPO group 1 |
| RMX80671.1 | Hortaea werneckii EXF-6656 | Ascomycetes | Fungi VHPO group 1 |
| RMY01705.1 | Hortaea werneckii EXF-6654 | Ascomycetes | Fungi VHPO group 1 |
| RMY07409.1 | Hortaea werneckii EXF-6669 | Ascomycetes | Fungi VHPO group 1 |
| RMY07560.1 | Hortaea werneckii EXF-6669 | Ascomycetes | Fungi VHPO group 1 |
| RMY14887.1 | Hortaea werneckii EXF-6654 | Ascomycetes | Fungi VHPO group 1 |
| RMY27915.1 | Hortaea werneckii EXF-6651 | Ascomycetes | Fungi VHPO group 1 |
| RMY49704.1 | Hortaea werneckii EXF-151 | Ascomycetes | Fungi VHPO group 1 |
| RMY78912.1 | Hortaea werneckii EXF-2682 | Ascomycetes | Fungi VHPO group 1 |
| RMZ13918.1 | Hortaea werneckii EXF-562 | Ascomycetes | Fungi VHPO group 1 |
| RMZ34245.1 | Hortaea werneckii EXF-120 | Ascomycetes | Fungi VHPO group 1 |
| XP_009227161.1 | Gaeumannomyces tritici | Ascomycetes | Fungi VHPO group 1 |
| XP_030982987.1 | Pyricularia grisea | Ascomycetes | Fungi VHPO group 1 |
| XP_003708974.1 | Pyricularia oryzae | Ascomycetes | Fungi VHPO group 1 |
| XP_003851923.1 | Zymoseptoria tritici | Ascomycetes | Fungi VHPO group 1 |
| XP_007759769.1 | Cladophialophora yegresii | Ascomycetes | Fungi VHPO group 1 |
| XP_008725073.1 | Cladophialophora carrionii | Ascomycetes | Fungi VHPO group 1 |
| CAA7260301.1 | Cyclocybe aegerita | Basidiomycetes | Fungi VHPO group 2 |
| KAF8229917.1 | Tricholoma matsutake | Basidiomycetes | Fungi VHPO group 2 |
| KAF9054890.1 | Panaeolus papilionaceus | Basidiomycetes | Fungi VHPO group 2 |
| KAG2019919.1 | Coprinopsis cinerea | Basidiomycetes | Fungi VHPO group 2 |
| KAH6905815.1 | Coprinopsis sp. | Basidiomycetes | Fungi VHPO group 2 |
| RPA75822.1 | Ascobolus immersus | Basidiomycetes | Fungi VHPO group 2 |
| TFK28780.1 | Coprinopsis marcescibilis | Basidiomycetes | Fungi VHPO group 2 |
| XP_001832980.2 | Coprinopsis cinerea | Basidiomycetes | Fungi VHPO group 2 |
| XP_028466698.1 | Sodiomyces alkalinus | Ascomycetes | Fungi VHPO group 2 |
| XP_001220510.1 | Chaetomium globosum | Ascomycetes | Fungi VHPO group 2 |
| XP_001800221.1 | Parastagonospora nodorum | Ascomycetes | Fungi VHPO group 2 |
| XP_001912717.1 | Podospora anserina | Ascomycetes | Fungi VHPO group 2 |
| XP_006695877.1 | Thermochaetoides thermophila | Ascomycetes | Fungi VHPO group 2 |
| XP_008085811.1 | Glarea lozoyensis | Ascomycetes | Fungi VHPO group 2 |
| XP_016643744.1 | Scedosporium apiospermum | Ascomycetes | Fungi VHPO group 2 |
| XP_024737652.1 | Hyaloscypha bicolor | Ascomycetes | Fungi VHPO group 2 |
| XP_033551804.1 | Lindgomyces ingoldianus | Ascomycetes | Fungi VHPO group 2 |
| 4USZ | Zobellia galactanivorans | Bacteroidetes | Flavobacteria vIPOs |
| WP_214610180.1 | Zobellia barbeyronii | Bacteroidetes | Flavobacteria vIPOs |
| WP_209402326.1 | Pseudozobellia sp. | Bacteroidetes | Flavobacteria vIPOs |
| WP_121850220.1 | Euzebyella marina | Bacteroidetes | Flavobacteria vIPOs |
| WP_138657405.1 | Maribacter algarum | Bacteroidetes | Flavobacteria vIPOs |
| 3W35 | Streptomyces sp. CNQ-525 | Actinobacteria | Actinobacteria vCPOs |
| WP_181785547.1 | Streptomyces sp. | Actinobacteria | Actinobacteria vCPOs |
| WP_181785550.1 | Streptomyces sp. | Actinobacteria | Actinobacteria vCPOs |
| WP_210581225.1 | Streptomyces sp. | Actinobacteria | Actinobacteria vCPOs |
| WP_230196915.1 | Streptomyces sp. | Actinobacteria | Actinobacteria vCPOs |

**Table S2.** Caver v1.1 pocket volume calculation in triplicates for each predicted *Hw*vCPO models and the *Ci*vCPO enzyme.

| Protein | Model | Pocket score (%) | Catalytic pocket volume triplicate (Å³) | Mean (Å³) |
| --- | --- | --- | --- | --- |
| *Hw*vCPO | 1 | 100 | 2558 | 2729 |
|  |  |  | 2775 |  |
|  |  |  | 2854 |  |
|  | 2 | 100 | 1734 | 1827 |
|  |  |  | 1865 |  |
|  |  |  | 1882 |  |
|  | 3 | 100 | 2233 | 2295 |
|  |  |  | 2316 |  |
|  |  |  | 2337 |  |
|  | 4 | 100 | 1970 | 2012 |
|  |  |  | 2006 |  |
|  |  |  | 2060 |  |
|  | 5 | 100 | 3992 | 4230 |
|  |  |  | 4344 |  |
|  |  |  | 4355 |  |
| *Ci*vCPO | NA | 100 | 1651 | 1774 |
|  |  |  | 1825 |  |
|  |  |  | 1845 |  |
